# Mapping SCA1 regional vulnerabilities reveals neural and skeletal muscle contributions to disease

**DOI:** 10.1172/jci.insight.176057

**Published:** 2024-03-21

**Authors:** Lisa Duvick, W. Michael Southern, Kellie A. Benzow, Zoe N. Burch, Hillary P. Handler, Jason S. Mitchell, Hannah Kuivinen, Udaya Gadiparthi, Praseuth Yang, Alyssa Soles, Carrie A. Sheeler, Orion Rainwater, Shannah Serres, Erin B. Lind, Tessa Nichols-Meade, Yun You, Brennon O’Callaghan, Huda Y. Zoghbi, Marija Cvetanovic, Vanessa C. Wheeler, James M. Ervasti, Michael D. Koob, Harry T. Orr

**Affiliations:** 1 Institute of Translational Neuroscience,; 2 Department of Laboratory Medicine and Pathology, and; 3 Department of Biochemistry, Molecular Biology, and Biophysics, University of Minnesota, Minneapolis, Minnesota, USA.; 4 Molecular Neurogenetics Unit, Center for Genomic Medicine, Massachusetts General Hospital, Boston, Massachusetts, USA.; 5 Department of Neuroscience, University of Minnesota, Minneapolis, Minnesota, USA.; 6 Mouse Genetics Laboratory, University of Minnesota, Minneapolis. Minnesota, USA.; 7 Departments of Molecular and Human Genetics, Pediatrics, and Howard Hughes Medical Institute, Baylor College of Medicine, Jan and Dan Duncan Neurological Research Institute at Texas Children’s Hospital, Houston, Texas, USA.; 8 Department of Neurology, Harvard Medical School, Boston, Massachusetts, USA.

**Keywords:** Neuroscience, Neurodegeneration

## Abstract

Spinocerebellar ataxia type 1 (SCA1) is a fatal neurodegenerative disease caused by an expanded polyglutamine tract in the widely expressed ataxin-1 (ATXN1) protein. To elucidate anatomical regions and cell types that underlie mutant ATXN1-induced disease phenotypes, we developed a floxed conditional knockin mouse (*f-ATXN1^146Q/2Q^*) with mouse *Atxn1* coding exons replaced by human *ATXN1* exons encoding 146 glutamines. *f-ATXN1^146Q/2Q^* mice manifested SCA1-like phenotypes including motor and cognitive deficits, wasting, and decreased survival. Central nervous system (CNS) contributions to disease were revealed using *f-ATXN1^146Q/2Q^*;*Nestin-Cre* mice, which showed improved rotarod, open field, and Barnes maze performance by 6–12 weeks of age. In contrast, striatal contributions to motor deficits using *f-ATXN1^146Q/2Q^*;*Rgs9-Cre* mice revealed that mice lacking *ATXN1^146Q/2Q^* in striatal medium-spiny neurons showed a trending improvement in rotarod performance at 30 weeks of age. Surprisingly, a prominent role for muscle contributions to disease was revealed in *f-ATXN1^146Q/2Q^*;*ACTA1-Cre* mice based on their recovery from kyphosis and absence of muscle pathology. Collectively, data from the targeted conditional deletion of the expanded allele demonstrated CNS and peripheral contributions to disease and highlighted the need to consider muscle in addition to the brain for optimal SCA1 therapeutics.

## Abstract 2

Targeted conditional deletion reveals that CNS regions and muscle contribute to spinocerebellar ataxia type 1 disease phenotypes.

## Graphical abstract

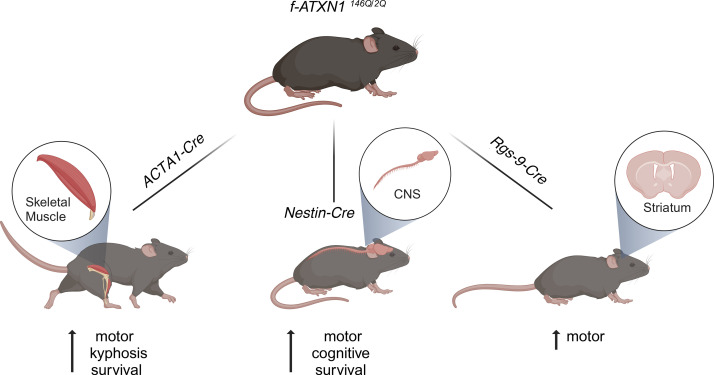

## Introduction

Neurodegenerative diseases are often characterized by a variety of clinical phenotypes. Thus, 2 fundamental issues facing neurodegenerative disease research are (a) the identification of the anatomical/cellular features underlying each disease symptom and (b) the assessment of the extent to which disease processes in these regions are similar at a molecular level. Progress in these areas provides vital information for developing treatments to ensure maximum therapeutic efficacy. In addition, mapping the anatomical basis of disease-associated phenotypes/symptoms is a means of further understanding CNS function at a systems level.

Spinocerebellar ataxia type 1 (SCA1), an autosomal dominant neurodegenerative disease, is one of the neurodegenerative diseases caused by expansion of a CAG trinucleotide tract that encodes a polyglutamine stretch in the resulting ataxin-1 (ATXN1) protein (1, 2). An initial symptom of SCA1 is typically gait ataxia. As the disease progresses, common symptoms include dysarthria, muscle wasting, and cognitive deficits such as executive functioning difficulties. Late-stage disease involves bulbar dysfunction, which is thought to cause the swallowing and breathing difficulties that underlie premature death (3–7). The typical pathological pattern is olivopontocerebellar atrophy with loss of cerebellar Purkinje cells, major neuronal loss in the dentate nuclei, and extensive olivary neuronal loss. Basal pontine neuronal atrophy is variable and, in some cases, severe. Other brainstem areas affected are the red nuclei, the vestibular nuclei, and motor cranial nerve nuclei (4, 7). In the spinal cord, the anterior horns, posterior columns, and spinocerebellar tracts also have atrophy with variable sparing of the pyramidal tracts. The pars compacta of the substantia nigra is relatively spared, but the striatum including the putamen, pallidum, and thalamus can have mild involvement. The basal forebrain cholinergic nuclei, cerebral cortex, and hippocampus may have mild neuronal loss (4, 7). Previous studies demonstrate the utility of *Atxn1-*knockin mice, in which an expanded stretch of CAG repeats is inserted into 1 *Atxn1* allele, as a model for studying the spectrum of SCA1-like phenotypes (8). In this study, we describe the generation and characterization of a SCA1–conditional knockin mouse model in which the 2 murine *Atxn1* coding exons, 7 and 8, in 1 allele were replaced with the 2 human *ATXN1* exons encoding the entire human ATXN1 protein — i.e., human exon 8, containing an expanded 146 CAG repeat, and human exon 9, which includes the stop codon and 3′UTR. LoxN recombination sites flanked the human *ATXN1* exons such that the human coding exons of the expanded *ATXN1^146Q/2Q^* allele were deleted in the presence of Cre recombinase. Heterozygous floxed humanized *ATXN1^146Q/2Q^* (*f-ATXN1^146Q/2Q^*) mice manifested a very similar collection of SCA1-like phenotypes as seen in *Atxn1^154Q^-*knockin mice (8) — i.e., a progressive neurological disorder featuring motor incoordination, cognitive deficits, wasting with kyphosis, and decreased survival. The large, expanded allele of both the original SCA1-knockin mouse and the one generated in this study render the mice an excellent and accurate genetic model for juvenile-onset SCA1, which typically shows much broader phenotypes but still allows studies of regional vulnerability to disease. To this end, we crossed *f-ATXN1^146Q/2Q^* mice with *Nestin-Cre* and *Rgs9*-*Cre* mice to reveal contributions of all CNS cells and striatal medium spiny neurons (MSNs) to SCA1-like phenotypes, respectively. Furthermore, *f-ATXN1^146Q/2Q^* were crossed to *ACTA1-Cre* mice, to assess the contribution of skeletal muscle to SCA1-like phenotypes. Using biochemical and image analyses comparing *f*-*ATXN1^146Q/2Q^;ACTA1-Cre* mice in which *f-ATXN1^146Q^* was deleted from muscle in *f-ATXN1^146Q/2Q^* mice, we showed that ATXN1[146Q] induced a strong peripheral muscle–specific phenotype. The findings from studying each of these subpopulations revealed that muscle involvement is prominent in this disease model while the striatal contribution is less prominent and might be a contributor in the late stage to disease progression.

## Results

### Generation and characterization of the conditional knockin f-ATXN1^146Q/2Q^ SCA1 mouse model.

**Figure 1:**
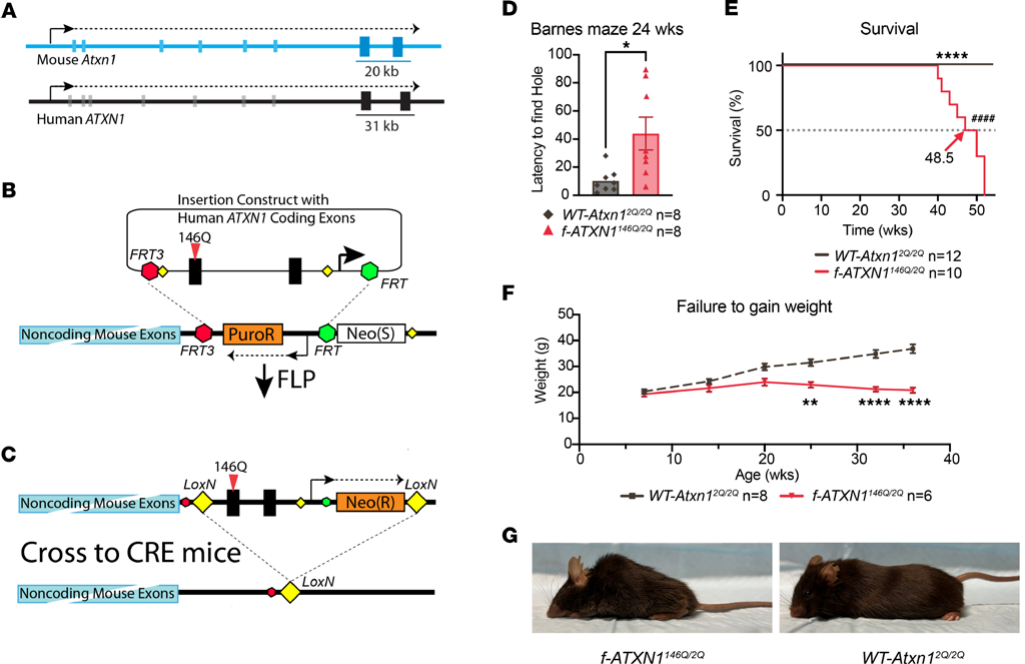
Generation and characterization of the *f-ATXN1^146Q/2Q^* conditional mouse model. (**A**) Organization of the mouse *WT-Atxn1* (blue) and human *ATXN1* (black) genes, with the only 2 exons encoding the ATXN1 protein indicated by boxes larger and darker than the noncoding exons. The size (kb) and location of the mouse genomic sequences (blue) replaced by the human genomic sequences (black) in the *f-ATXN1^146Q^* allele are indicated. (**B**) The portion of the *Atxn1* gene encompassing the 2 coding exons was replaced with an FRT-recombination recipient cassette in mouse ES cells; then, that cassette was replaced with the portion of the human *ATXN1* genomic sequences syntenic to the deleted mouse sequence using FLP recombinase. (**C**) The inserted human sequences in the resulting *ATXN1^146Q^* allele are flanked by LOX recombination sites, as shown. Mating mice with this allele to lines expressing CRE recombinase removes the human *ATXN1* insertion, as shown. (**D**) Barnes maze performance at 24 weeks of age, using unpaired 2-tailed *t* test. (**E**) Mouse survival plotted as Kaplan-Meier curves with median lifespan labeled for each genotype. Log-rank (Mantel Cox) ****P < 0.0001 and Gehan-Breslow-Wilcoxon ^####^*P* < 0.0001. (**F**) Body weight measurements between 6 and 36 weeks of age, repeated-measures 2-way ANOVA with Geisser-Greenhouse correction and Šídák’s post hoc test. (**G**) Representative photographs of 42-week-old *WT-Atxn1^2Q/2Q^* and *f-ATXN1^146Q/2Q^* showing kyphosis. *P < 0.05, **P < 0.01, and ****P < 0.0001.

A conditional knockin mouse model designated *f*-*ATXN1^146Q/2Q^* was developed to investigate brain region and peripheral tissue contributions to SCA1-like phenotypes as well as to obtain a platform for testing human *ATXN1* gene targeting therapies. The entire coding region of 1 allele of the mouse *Atxn1* gene was replaced with that of the human *ATXN1* gene in a 2-step process. First, the 3′ end of the *Atxn1* gene (20 kb), including the last 2 exons through the end of the transcript (Figure 1A), was deleted from the genome of a mouse ES cell line (C57BL/6N) and replaced by flippase recognition target (FRT) recombination sites and selectable markers (Figure 1B). Next, the syntenic sequence from the human genome (31 kb) was inserted using site-specific recombination at flanking FRT sites (Figure 1C). The ES cell was injected into C57BL/6N embryos yielding *f-ATXN1*^146Q/–^ mice, which were then crossed with WT mice to yield *f-ATXN1^146Q/2Q^* mice. With respect to coding sequences, these mice have 1 mouse WT allele and 1 disease-causing human allele encoding ATXN1 containing 146 glutamine repeats.

To evaluate the ability of the *ATXN1^146Q^* allele to be deleted by Cre recombinase, *f-ATXN1^146Q/2Q^* mice were crossed with *Sox2-Cre* mice (9). This *Sox2-Cre* mouse mediates efficient Cre-mediated recombination at gastrulation in all epiblast-derived cells including the primordial germ cells. Using CAG repeat-specific and recombination-specific PCR primers (Supplemental Figure 1A; supplemental material available online with this article; https://doi.org/10.1172/jci.insight.176057DS1), recombination and deletion of the *ATXN1^146Q^* allele was detected in all tissues examined from a *f-ATXN1^146Q/2Q^;Sox2-Cre* mouse (Supplemental Figure 1, B–D).

Expression of the ATXN1[146Q] protein was assessed by immunoblotting protein extracts prepared from 3 brain regions from *WT-Atxn1^2Q/2Q^*, *f-ATXN1^146Q/2Q^*, and *Atxn1^175Q/2Q^* mice, the latter derived from the *Atxn1^154Q/2Q^* knockin line developed by Watase et al. (8). In 2-week-old mice, the levels of ATXN1[146Q] and ATXN1[175Q] relative to mouse ATXN1[2Q] or TUBULIN were similar in the 3 brain regions examined: cerebral cortex, cerebellum, and brainstem (Supplemental Figure 2, A–C). Expression of the *f-ATXN1^146Q^* transcript was also examined by quantitative PCR (qPCR) (Supplemental Figure 2D). Total RNA was extracted from the cerebellum of 2-week-old *f-ATXN1^146Q/2Q^* mice. Human *f-ATXN1^146Q^* RNA levels are not statistically different from mouse *WT-Atxn1^2Q^* RNA levels in the cerebellum (Supplemental Figure 2E).

*f-ATXN1^146Q/2Q^* mice display the spectrum of SCA1-like phenotypes observed in *Atxn1^154Q/2Q^* mice (8) and, due to the large repeat expansion, recapitulate those seen in juvenile patients (10). The SCA-like phenotypes exhibited by *f-ATXN1^146Q/2Q^* mice include a cognitive deficit on the Barnes maze (Figure 1D), reduced survival (Figure 1E), and wasting (Figure 1F). In addition, *f-ATXN1^146Q/2Q^* mice also have prominent kyphosis (Figure 1G). Typical of SCA1 mouse models, *f-ATXN1^146Q/2Q^* mice have a motor performance deficit as assessed by an accelerating rotarod assay (see below).

### Affect of deleting the ATXN1^146Q^ allele from the CNS on SCA1-like phenotypes.

**Figure 2:**
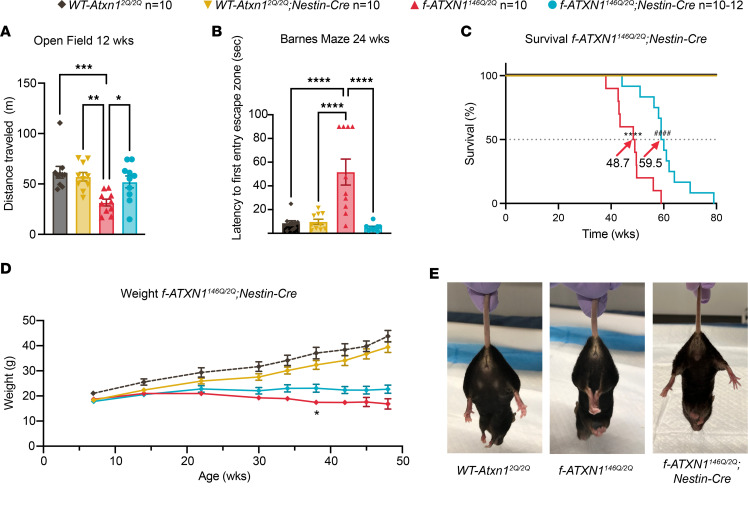
SCA1-like phenotypes improved in *f-ATXN1^146Q/2Q^;Nestin-Cre* mice. (**A**) Open-field performance at 12 weeks of age, using 1-way ANOVA Tukey’s post hoc test. (**B**) Barnes maze performance at 24 weeks of age, using 1-way ANOVA Tukey’s post hoc test. (**C**) Mouse survival plotted as Kaplan-Meier curves with median lifespan labeled for each genotype. Log-rank (Mantel Cox) ****P < 0.0001 and Gehan-Breslow-Wilcoxon ^####^*P* < 0.0001. (**D**) Body weight measurements between 7 and 48 weeks of age. Significant weight difference shown is between *f-ATXN1^146Q/2Q^* and *f-ATXN1^146Q/2Q^;Nestin-Cre* at 38 weeks. Repeated-measures 2-way ANOVA Geisser-Greenhouse correction and Tukey’s post hoc test. (**E**) Hind limb clasping at 36 weeks. *P < 0.05, **P < 0.01, ***P < 0.001, and ****P < 0.0001.

*Nestin-Cre* mice express Cre recombinase in neuronal and macroglial precursors and, thus, throughout the CNS (11). *f-ATXN1^146Q/2Q^;Nestin-Cre* mice were examined to assess the extent to which deletion of the *f-ATXN1^146Q^* allele from CNS neuronal and glial cells improved other SCA1-like neurological phenotypes manifested by *f-ATXN1^146Q^* mice. *f-ATXN1^146Q/2Q^;Nestin-Cre* mice had a remarkable and significant improvement in performance in the open-field test (locomotion) compared with *f-ATXN1^146Q/2Q^* mice at 12 weeks of age (Figure 2A). *f-ATXN1^146Q/2Q^;Nestin-Cre* mice also had normalized cognitive performance, as evaluated by the Barnes maze test. Compared with controls *WT-Atxn1^2Q/2Q^* and *WT-Atxn1^2Q/2Q^;Nestin-Cre* mice, *f-ATXN1^146Q/2Q^* mice showed a significant delay in their first entry into the escape hole zone, while *f-ATXN1^146Q/2Q^;Nestin-Cre* mice did not (Figure 2B). *f-ATXN1^146Q/2Q^;Nestin-Cre* mice had significant improvement in survival (Figure 2C) and a small but trending improvement in weight compared with *f-ATXN1^146Q/2Q^*, significant only for mice at 38 weeks. Overall, their weight remained reduced compared with WT mice (Figure 2D). In addition, at 36 weeks of age, *f-ATXN1^146Q/2Q^* displayed a hindlimb clasping phenotype characteristic of mice with pathology in a variety of brain regions (Figure 2E) (12). The hindlimb clasping phenotype was absent in *f-ATXN1^146Q/2Q^;Nestin-Cre* mice at this age.

**Figure 3:**
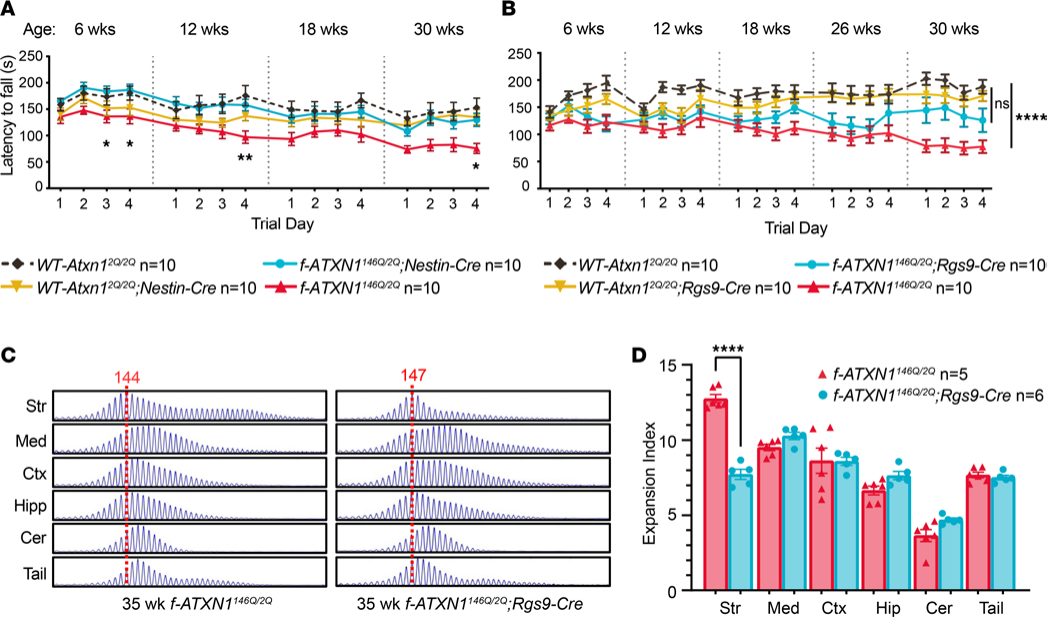
Striatal pathology contributes to the progressive motor performance deficit in *f-ATXN1^146Q/2Q^* mice. (**A**) Rotarod assessment: *WT-Atxn1^2Q/2Q^*, *WT-Atxn1^2Q/2Q^;Nestin-Cre*, *f-ATXN1^146Q/2Q^;Nestin-Cre*, and *f-ATXN1^146Q/2Q^* at 6, 12, 18, and 30 weeks. (**B**) Rotarod assessment: *WT-Atxn1^2Q/2Q^*, *WT-Atxn1^2Q/2Q^;Rgs9-Cre, f-ATXN1^146Q/2Q^;Rgs9-Cre*, and *f-ATXN1^146Q/2Q^* at 6, 12, 18, 26, and 30 weeks. Statistics shown on the graph for **A** represent statistical significance between *f-ATXN1^146Q/2Q^* and *f-ATXN1^146Q/2Q^;Nestin-Cre*; repeated-measures 2-way ANOVA with Geisser-Greenhouse correction and Tukey’s post hoc test were performed separately for each time point. For **B**, repeated-measures 2-way ANOVA with Geisser-Greenhouse correction showing significance between *WT-Atxn1^2Q/2Q^* and *f-ATXN1^146Q/2Q^* and not between *WT-Atxn1^2Q/2Q^* and *f-ATXN1^146Q/2Q^;Rgs9-Cre*. *P < 0.05, **P < 0.01, and ****P < 0.0001. (**C**) Representative GeneMapper traces showing somatic instability in 35-week *f-ATXN1^146Q/2Q^* and *f-ATXN1^146Q/2Q^;Rgs9-Cre* mice. (**D**) Quantified expansion indices. *f-ATXN1^146Q/2Q^*, CAG 142–147 (determined from stable striatal peak [red dotted line]); *f-ATXN1^146Q/2Q^;Rgs9-Cre*, CAG 145–138. Unpaired 2-tailed *t* test. ****P < 0.0001.

Naive *f-ATXN1^146Q/2Q^* mice have a significant deficit in performance on the accelerating rotarod at 6 weeks that progresses in severity with age (Figure 3, A and B, and Supplemental Figure 4). Notably, *f-ATXN1^146Q/2Q^;Nestin-Cre* mice with the *ATXN1^146Q^* allele deleted throughout the CNS (11) displayed improved rotarod performance, practically to WT levels, at all ages tested, from 6 to 30 weeks of age (Figure 3A and Supplemental Figure 4B). Previous studies in which the cerebella of 5-week-old SCA1-knockin mice were injected with adeno-associated viruses expressing inhibitory RNAs targeting *Atxn1* showed rescued rotarod performance, indicating a critical role of the cerebellum in the motor coordination deficit seen in SCA1 (13, 14). Of note, in patients with SCA1, changes in striatal volume are associated with an age-associated decline in motor performance (15, 16). Thus, as a first step in assessing regions of the CNS, in addition to the cerebellum where dysfunction might contribute to altered rotarod performance with increasing age, *f-ATXN1^146Q/2Q^* mice were crossed to *Rgs9-Cre* mice to delete the *ATXN1^146Q^* allele from striatal MSNs (Figure S5) (17, 18). Interestingly, deletion of *ATXN1^146Q^* from striatal MSNs trended toward partial improvement of rotarod performance that did not manifest until 30 weeks of age in *f-ATXN1^146Q/2Q^;Rgs9-Cre* mice relative to *f-ATXN1^146Q/2Q^* mice (Figure 3B and Supplemental Figure 4C). While *f-ATXN1^146Q/2Q^* mice significantly declined over time, *ATXN1^146Q/2Q^;Rgs9-Cre* were not significantly different compared with *WT-Atxn1^2Q/2Q^* or *WT-Atxn1^2Q/2Q^;Rgs9-Cre* mice. Deletion of *ATXN1^146^* from striatal MSNs significantly improved dopamine- and cAMP-regulated phosphoprotein-32 (DARPP-32) expression in the striatum of *f-ATXN1^146Q/2Q^;Rgs9-Cre* mice (Supplemental Figure 6, A and B).

Previous studies show extensive somatic expansion of the *ATXN1* CAG repeat in SCA1 patient and knockin mice striatum (19, 20). Analyses of 35-week *f-ATXN1^146Q/2Q^* mice also revealed high levels of somatic expansion in striatum. CAG expansion was also observed in other tissues/brain regions, most notably the medulla (Figure 3, C and D), with *f-ATXN1^146Q/2Q^* mice exhibiting a brain regional expansion profile similar to that of a patient with SCA1 (20). Striatal expansion was significantly reduced in *f-ATXN1^146Q/2Q^;Rgs9-Cre* mice (Figure 3, C and D), demonstrating that expansions occur in MSNs. Residual striatal expansion in *f-ATXN1^146Q/2Q^;Rgs9-Cre* mice may be explained by incomplete Cre-mediated excision of the expanded CAG repeat-containing allele in MSNs and/or CAG expansion in other striatal cell types (Supplemental Figure 5). We speculate that expansion of expanded *ATXN1* CAG in the striatum contributes to mild deficits in motor performance with disease progression in *f-ATXN1^146Q/2Q^* mice. That CAG expansion occurs in the striatal MSNs is interesting. However, the data indicate that such expansions may contribute to striatal pathogenesis only late in the course of disease.

### Deletion of the f-ATXN1^146Q^ allele from skeletal muscle reveals a direct muscle-specific pathogenic effect of ATXN1[146Q].

**Figure 4:**
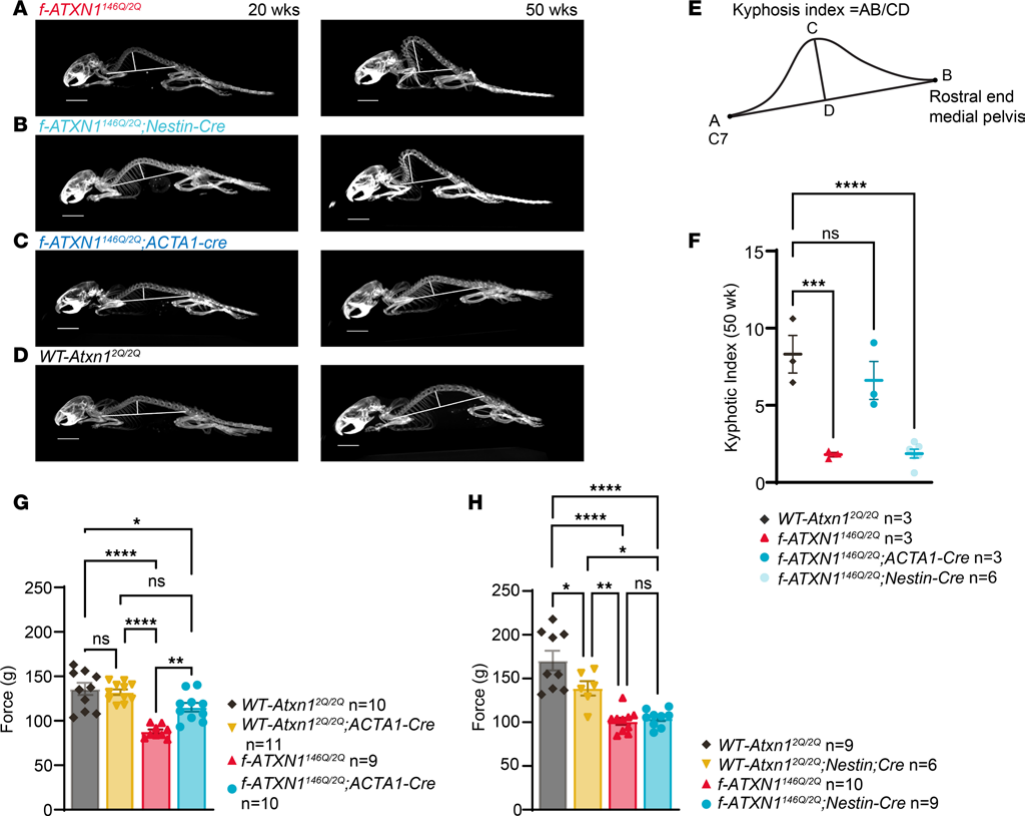
Progression of kyphosis pathology in *f-ATXN1^146Q/2Q^* mice is corrected with deletion of ATXN1^146Q^ in skeletal muscle. (**A**–**D**) CT scan image of *f-ATXN1^146Q/2Q^*, *f-ATXN1^146Q/2Q^;Nestin-Cre*, *f-ATXN1^146Q/2Q^;ACTA1-Cre*, and *WT-Atxn1^2Q/2Q^* mice at 20 and 50 weeks. Scale bar: 10 mm. (**E** and **F**) Kyphosis image calculation and kyphotic index at 50 weeks, using 1-way ANOVA, compared with *WT-Atxn1^2Q/2Q^* with Dunnett’s post hoc test. ***P < 0.001 and ****P < 0.0001. (**G**) Assessment of grip strength for *f-ATXN1^146Q/2Q^* and *f-ATXN1^146Q/2Q^;ACTA1-Cre* compared with *WT-Atxn1^2Q/2Q^* and *WT-Atxn1^2Q/2Q^;ACTA1-Cre* mice at 18 weeks of age. (**H**) Assessment of grip strength for *f-ATXN1^146Q/2Q^* and *f-ATXN1^146Q/2Q^;Nestin*-*Cre* compared with *WT-Atxn1^2Q/2Q^* and *WT-Atxn1^2Q/2Q^;Nestin-Cre* mice at 18 weeks of age. One-way ANOVA with Tukey’s post hoc test were performed for all. *P < 0.05, **P < 0.01, and ****P < 0.0001.

It has been noted that muscle wasting is observed in patients with SCA1, and in mice, muscle myopathy could be contributing to kyphosis (21). Figure 4A shows that *f-ATXN1^146Q/2Q^* mice manifest a severe kyphosis by 20 weeks of age with increasing severity to 50 weeks of age. We examined the CNS effect of ATXN1[146Q] on kyphosis by deleting ATXN1[146Q] from all CNS neurons, including spinal motor neurons, by crossing *f-ATXN1^146Q/2Q^* mice with *Nestin-Cre* mice. Image analysis showed that kyphosis in *f-ATXN1^146Q/2Q^;Nestin-Cre* mice remained severe (Figure 4B). To examine whether kyphosis in *f-ATXN1^146Q/2Q^* mice is due to a direct effect of ATXN1[146Q] on muscle, *f-ATXN1^146Q/2Q^* mice were crossed with transgenic *ACTA1-Cre* *(HSA-Cre79)* mice to delete the *ATXN1^146Q^* allele from muscle (22). In *f-ATXN1^146Q/2Q^;ACTA1-Cre* mice, expression of the *ATXN1^146Q^* allele was significantly reduced in skeletal muscle, including the tongue and diaphragm but not in the heart, cerebral cortex, cerebellum, medulla, or lung (Supplemental Figure 3, C and E). *ACTA1-Cre*–induced deletion of *ATXN1^146Q^* eliminated the kyphosis seen in *f-ATXN1^146Q/2Q^* mice, restoring the spinal configuration similar to that seen in WT mice at 20 and 50 weeks of age (Figure 4, C and D). Using an approach to calculate a kyphosis index (KI), the KI of *f-ATXN1^146Q/2Q^* mice is significantly reduced compared with the KI of *WT-Atxn1^2Q/2Q^* mice (Figure 4, E and F). Notably, deletion of the *ATXN1^146Q^* allele from muscle restored the KI to that of *WT-Atxn1^2Q/2Q^* mice. In addition, the grip strength test used to measure the neuromuscular function as maximal muscle strength showed significant rescue when *ATXN1^146Q^* was deleted from muscle in the *ACTA1-Cre* mice but not the *Nestin-Cre* mice (Figure 4, G and H). Interestingly, there was a slight but significant difference between the *WT-Atxn1^2Q/2Q^* versus *WT-Atxn1^2Q/2Q^*; *Nestin-Cre* mice in grip strength at 18 weeks. Of note, the *Nestin-Cre* transgenic line trended slighter smaller (Figure 2D), presumably due to this Cre line being affected by mild hypopituitarism, which could impair grip strength (23). These data support the concept that kyphosis and the deficit in grip strength in *f-ATXN1^146Q/2Q^* mice are the result of muscle dysfunction induced by expression of ATXN1[146Q] in muscle.

**Figure 5:**
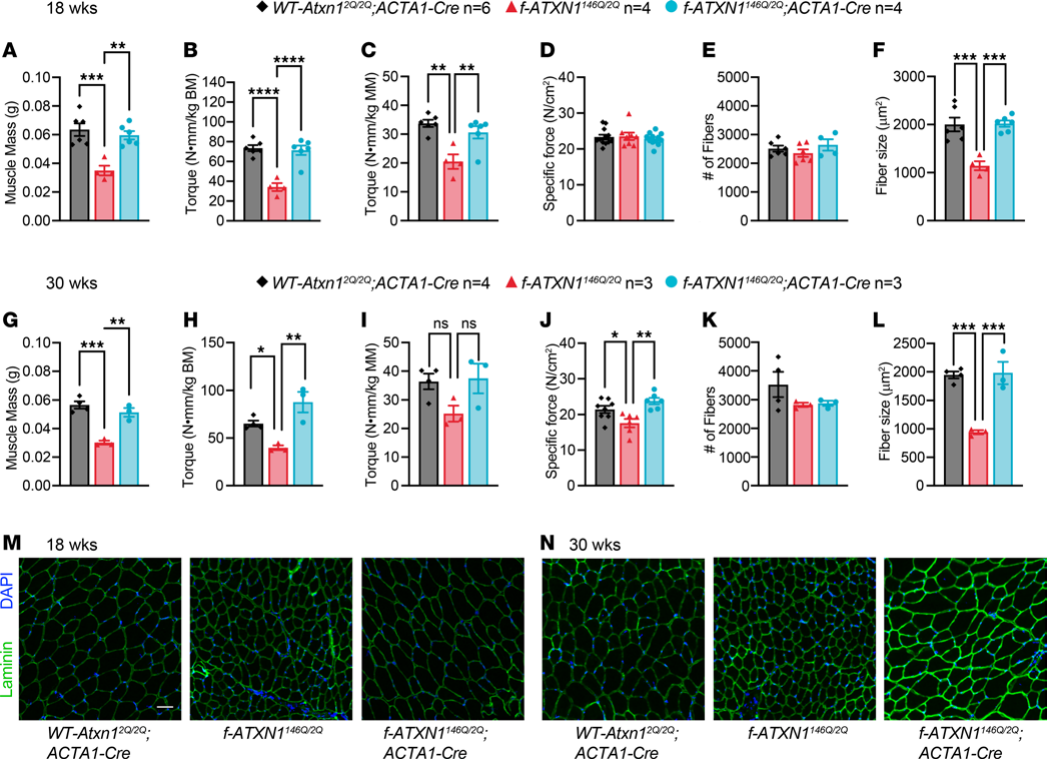
Skeletal muscle pathology in *f-ATXN1^146Q/2Q^* mice is corrected with deletion of the *ATXN1^146Q^* allele. (**A** and **G**) Dorsiflexor muscle mass comprised of the sum of tibialis anterior (TA) and extensor digitorum longus (EDL) muscle masses for 18- and 30-week-old *WT-Atxn1^2Q/2Q^;ACTA1-Cre*, *f-ATXN1^146Q/2Q^*, and *f-ATXN1^146Q/2Q^;ACTA1-Cre* mice. (**B**, **C**, **H**, and **I**) Peak anterior crural isometric torque at 18 and 30 weeks normalized to body mass and dorsiflexor mass. (**D** and **J**) Specific isometric force from ex vivo preparations of EDL muscles at 18 and 30 weeks. (**M** and **N**) Representative cross-sectional images from TA muscles showing nuclear (blue; DAPI) and laminin (green) staining at 18 and 30 weeks. Scale bar: 50 μm. (**E** and **K**) Quantification of total number of fibers at 18 and 30 weeks. (**F** and **L**) Average fiber size for TA muscle cross-sections at 18 and 30 weeks. One-way ANOVA with Tukey’s post hoc test were performed for all except **M** and **N**. *P < 0.05, **P < 0.01, ***P < 0.001, and ****P < 0.0001.

**Figure 6:**
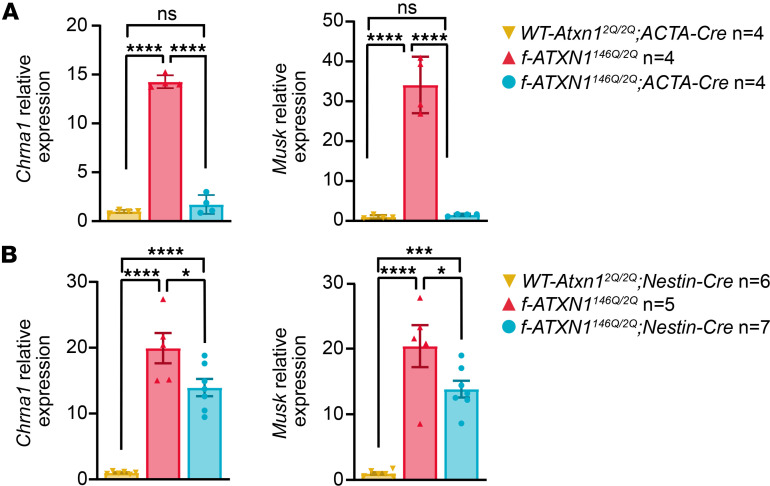
Expression of *Chrna1* and *Musk* encoding NMJ proteins is altered in *f-ATXN1^146Q/2Q^* muscle. (**A**) Relative expression of *Chrna1* and *Musk* in 40-week quadricep RNA from *WT-Atxn1^2Q/2Q^;ACTA1-Cre*, *f-ATXN1^146Q/2Q^*, and *f-ATXN1^146Q/2Q^;ACTA1-Cre* mice. (**B**) Relative expression of *Chrna1* and *Musk* in 40-week quadricep RNA from *WT-Atxn1^2Q/2Q^;Nestin-Cre*, *f-ATXN1^146Q/2Q^*, and *f-ATXN1^146Q/2Q^;Nestin-Cre* mice. Expression is normalized to the average of *Gapdh* and *Actb*. One-way ANOVA with Tukey’s post hoc test were performed for all. *P < 0.05, ***P < 0.001, and ****P < 0.0001.

Consistent with an ATXN1[146Q]-induced muscle phenotype, tibialis anterior (TA) and extensor digitorum longus (EDL) muscle masses were lower in *f-ATXN1^146Q/2Q^* mice compared with *WT-Atxn1^2Q/2Q^*; *ACTA1-Cre* or *f-ATXN1^146Q/2Q^*;*ACTA1-Cre* mice at both 18 and 30 weeks (Figure 5, A and G). Additional characterization of the effects of ATXN1[146Q] on skeletal muscle function revealed a progressive, muscle-specific myopathy. Further evaluation of muscle strength was performed using an in vivo electrode-based assay that measures dorsiflexor muscle torque via direct stimulation of the peroneal nerve. Dorsiflexor (EDL + TA) torque was significantly lower at 18 weeks and trending lower at 30 weeks of age in *f-ATXN1^146Q/2Q^* mice compared with *WT-Atxn1^2Q/2Q^*;*ACTA1-Cre* mice, despite correcting for body mass or muscle mass (Figure 5, B, C, H, and I). To further investigate the ATXN1[146Q]-induced muscle weakness phenotype, we used an ex vivo assay that assesses EDL contractile function independent of endogenous motor neuron activation. At 18 weeks of age, EDL specific force production was not different between genotypes, suggesting that intrinsic muscle contractility, independent of motor neuronal activation, was not affected in *f-ATXN1^146Q/2Q^* mice (Figure 5D). However, at 30 weeks, EDL specific force was lower in the *f-ATXN1^146Q/2Q^* mice, a phenotype that was fully corrected with deletion of the *ATXN1^146Q^* allele from muscle (Figure 5J). The in vivo but not ex vivo muscle strength deficits in *f-ATXN1^146Q/2Q^* mice at 18 weeks suggest the presence of a neuronal pathology potentially related to disrupted motor neuron activation or neuromuscular junction (NMJ) dysfunction. Consistent with possible alterations in NMJ function, at 8 weeks of age, increased expression of mRNAs encoding the NMJ proteins MuSK and CHRNA1 (24) were detected (Supplemental Figure 6C). At 40 weeks of age, expression of *Musk* and *Chrna1* RNAs were restored to normal levels in *f-ATXN1^146Q/2Q^*;*ACTA1-Cre* mice (Figure 6A) but not in *f-ATXN1^146Q/2Q^;Nestin-Cre* mice (Figure 6B). Furthermore, the reduction in EDL specific force observed at 30 weeks in *f-ATXN1^146Q/2Q^* mice could indicate a progressive myopathy stemming from a combination of chronic muscle disuse and alterations in intrinsic muscle contractile function. Importantly, all muscle deficits were restored to *WT-Atxn1^2Q/2Q^;ACTA1-Cre* levels in mice with the *ACTA1-Cre–*induced deletion of the *ATXN1^146Q^* allele. Together, these results indicate the presence of a progressive myopathy that eventually affects intrinsic muscle function.

Histological analysis of muscle cross sections at 18 and 30 weeks revealed that total fiber number of the TA was not different between genotypes but that the *f-ATXN1^146Q/2Q^* TA muscles had significantly smaller fiber cross-sectional area compared with *WT-Atxn1^2Q/2Q^;ACTA1-Cre* TA muscles (Figure 5, E, F, and K–N). Muscle fiber size was completely rescued in *f-ATXN1^146Q/2Q^*;*ACTA1-Cre* mice (Figure 5, F and L). These data support the presence of a *f-ATXN1^146Q/2Q^* specific skeletal muscle pathology that is characterized by muscle weakness and smaller muscle size.

**Figure 7:**
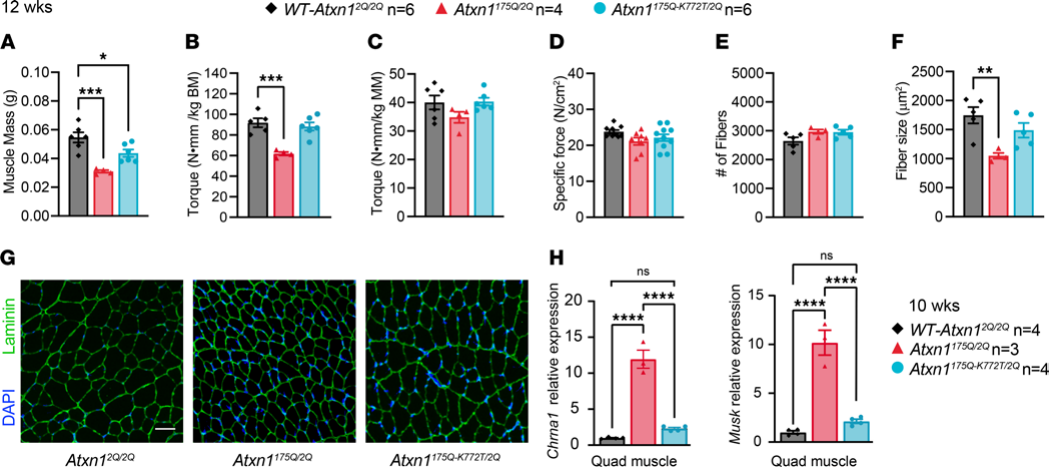
Nuclear localization of expanded ATXN1 is important for muscle-specific phenotypes caused by expanded ATXN1. (**A**) Dorsiflexor muscle mass composed of the sum of tibialis anterior (TA) and extensor digitorum longus (EDL) muscle masses for 12-week-old *WT-Atxn1^2Q/2Q^*, *Atxn1^175Q/2Q^,* and *Atxn1^175Q–K772T/2Q^* mice. (**B** and **C**) Peak anterior crural isometric torque normalized to body mass (**B**) and dorsiflexor mass (**C**). (**D**) Specific isometric force from ex vivo preparations of EDL muscles. (**E** and **F**) Quantification of total number of fibers (**E**) and average fiber size (**F**) from TA muscle cross-sections. Scale bar: 50 μm. (**G**) Representative cross-sectional images from TA muscles showing nuclear (blue; DAPI) and laminin (green) staining. (**H**) Relative expression of *Chrna1* or *Musk* normalized to the average of *Gapdh* and *Actb* in 10-week quadricep RNA from *WT-Atxn1^2Q/2Q^*, *Atxn1^175Q/2Q^*, and *Atxn1^175Q–K772T/2Q^* mice. One-way ANOVA with Tukey’s post hoc test were performed for all except **G**. *P < 0.05, **P < 0.01, ***P < 0.001, and ****P < 0.0001.

Proper nuclear localization of mutant ATXN1 is critical for many disease-like phenotypes, including motor dysfunction, cognitive deficits, and premature lethality (25). Like in *f-ATXN1^146Q/2Q^* mice, smaller dorsiflexor muscle mass and torque (Figure 7, A–C) and smaller fiber size (Figure 7F), indicating the presence of a skeletal muscle myopathy, are seen in *Atxn1^175Q/2Q^* mice as early as 12 weeks of age. As in *f-ATXN1^146Q/2Q^* mice, *Atxn1^175Q/2Q^* mice had similar specific force and number of muscle fibers as *WT-Atxn1^2Q/2Q^* mice (Figure 7, D and E). Notably, this phenotype was partially corrected in *Atxn1^175Q–K772T/2Q^* mice in which ATXN1’s nuclear localization sequence is disrupted. The *Atxn1^175Q–K772T/2Q^* muscles demonstrate partially recovered muscle mass (Figure 7A) and muscle fiber size (Figure 7, F and G). As in *f-ATXN1^146Q/2Q^;ACTA-Cre* mice, expression of NMJ genes were rescued to *WT-Atxn1^2Q/2Q^* levels in *Atxn1^175Q-K772T/2Q^* mice (Figure 7H). Thus, as with the other SCA1-like disease phenotypes, we conclude that nuclear localization of expanded ATXN1 is important for the muscle-specific phenotypes caused by expanded ATXN1.

Deleting the *f-ATXN1^146Q^* allele from muscle in the *f-ATXN1^146Q/2Q^*;*ACTA1-Cre* mice showed no improvement in open field, rotarod, or hind limb clasping phenotypes (Supplemental Figure 7, A–C). In contrast, survival and wasting were significantly improved in *f-ATXN1^146Q/2Q^*;*ACTA1-Cre* mice (Supplemental Figure 7, D and E), supporting the concept that mutant ATXN1-induced muscle pathology contributes to these SCA1-like phenotypes.

## Discussion

Neurodegenerative diseases are typically characterized by prominent pathology in specific brain regions/neuronal populations; however, many of these disorders affect other neural structures, circuits, and cell populations that lack obvious pathology. In SCA1, cerebellar Purkinje cell degeneration is a frequent and prominent pathological feature. However, ATXN1 is widely expressed, and patients with SCA1 present with symptoms linked to multiple different brain regions (7). In particular, individuals with large repeat expansion and juvenile onset have broader phenotypes, including cognitive deficits, striatal dysfunction, and muscle wasting (10), highlighting that, as the repeat grows, more and more cells become vulnerable to disease. A question facing the understanding of SCA1 pathogenesis, as well as many other neurodegenerative diseases, is the extent to which vulnerability in specific cell populations contributes to each disease-associated phenotype. In this study, we addressed this question in SCA1 using a conditional mouse model with broad spatial mutant gene expression that matches that of humans. We found that deletion of the *f-ATXN1^146Q^* allele from CNS neuronal and glial cells rescued SCA1-like neurological phenotypes seen in the *f-ATXN1^146Q/2Q^* mice. Analysis of a key disease phenotype, deficit in motor performance on the rotarod, showed that this phenotype is very nicely rescued in *Nestin-Cre* mice and that it is predominantly of cerebellar origin because rescue in striatal MSNs was minimal and only very late in disease course. Additionally, we found direct pathological effects of *f-ATXN1^146Q^* on muscle and pinpointed that this contributes to wasting and kyphosis.

Like the *Atxn1^154Q/2Q^-*knockin mice (8), *f-ATXN1^146Q/2Q^* mice developed neurological phenotypes similar to those seen in patients with SCA1. Motor incoordination on the rotarod was seen as early as 7 weeks of age in *f-ATXN1^146Q/2Q^* mice, and cognitive deficits became apparent as assessed by the Barnes maze test later at 24 weeks of age. Confirming the neural basis of these SCA1-like phenotypes, substantial rescue of rotarod performance (Figure 3A and Supplemental Figure 4B), performance in the open field test (Figure 2A) and on the Barnes maze (Figure 2B) was observed in *f-ATXN1^146Q/2Q^* mice crossed to *Nestin-Cre* mice. The *Nestin-Cre* line was designed to direct Cre recombinase expression to neuronal and macroglial precursors, providing recombination exclusively in the CNS (11). Analysis of the tissue pattern of *f-ATXN1^146Q^* deletion in *f-ATXN1^146Q/2Q^;Nestin-Cre* mice confirmed the CNS specificity of *Nestin-Cre* recombination (Supplemental Figure 3, A, B, and D). As an initial effort to further dissect the anatomical basis of a key SCA1-like phenotype manifested by the *f-ATXN1^146Q/2Q^* mice, we selected motor incoordination as measured by rotarod performance. Interestingly, while injection of an *Atxn1-*targeting siRNA into the cerebellum was able to improve rotarod performance at 10 weeks of age in *Atxn1^154Q/2Q^-*knockin mice (13), deletion of *f-ATXN1^146Q^* from the striatum by crossing *f-ATXN1^146Q/2Q^* mice to *Rgs9-Cre* mice decreased the decline in rotarod performance seen late in disease progression at 30 weeks of age. Correspondingly at diagnosis, magnetic resonance imaging (MRI) of patients with SCA1 shows that loss of cerebellar volume is essentially complete. As patients with SCA1 age, MRI analyses show a progressive loss in striatal volume (15, 16). Moreover, a recent study found striatal volume to be a predictor of motor decline with increasing patient age after onset of ataxia (16). Our findings indicate that a relative time course of disease in cerebellum and striatum in *f-ATXN1^146Q/2Q^* mice parallel the MRI findings in patients with SCA1.

Previous studies show that, in the striatum, somatic instability of expanded *ATXN1* and *HTT* are similar to each other in knockin mice and patients (19, 20, 26). Here, we show that, in *f-ATXN1^146Q/2Q^* mice, striatal *ATXN1* CAG expansion occurs in MSNs (Figure 3, C and D). In contrast to HD where repeat expansion in striatal MSNs drives onset of disease, data presented here along with previous data indicate that, in SCA1, expanded ATXN1 expression in the cerebellum drives disease onset and that striatal ATXN1 repeat expansion only contributes to age-dependent progression of motor deficits well after disease onset (Figure 3B and Supplemental Figure 4C). Thus, we reason that somatic instability alone does not drive HD and SCA1, and we reason that the protein context and corresponding function of the protein in which the repeat expansion occurs is critical for driving the course of disease pathogenesis.

As disease progresses in patients with SCA1, muscle wasting is often seen (27–30). Signs of motor neuron pathology were suggested as being linked to muscle wasting (31). In this study, we provide evidence that expanded ATXN1 has a direct pathological effect on skeletal muscles. At 18 weeks, we observed that in vivo torque deficits were not corrected even after normalizing muscle strength by muscle size. This suggests a muscle force–generating pathology that could be stemming from a variety of muscle contraction–related elements ranging from motor neuron activation to myosin-actin cross bridge cycling. Interestingly, ex vivo force at 30 weeks was significantly lower in *f-ATXN1^146Q/2Q^* mice than *WT-Atxn1^2Q/2Q;^ACTA1-Cre* mice, which suggests that the muscle pathology could progress to more specific disruptions in intrinsic contractile function. Notably, every muscle deficit was restored to that seen in *WT-Atxn1^2Q/2Q^* mice in *f-ATXN1^146Q/2Q^* mice in which *ACTA1-Cre* induced deletion of the *ATXN1^146Q^* allele. Overall, the data point to the presence of a neuromuscular pathology perhaps involving dysfunction at the NMJ. That NMJ function is impaired in *f-ATXN1^146Q^* mice is supported by the finding that expression of *Musk* and *Chrna1* RNA is altered in muscle of *f-ATXN1^146Q^* mice. Further investigation is necessary to explore the contractile-specific effects of the muscle-specific ATXN1[146Q] mutation.

Notably, deletion of *f-ATXN1^146Q^* from muscle substantially reduced kyphosis — which, in mice, can be associated with paraspinal skeletal muscle impairment (21) — as well as restored normal dorsiflexor strength, muscle mass and fiber size. As seen for other SCA1-like phenotypes, motor dysfunction, cognitive deficits, and premature lethality (25), proper nuclear localization of expanded ATXN1 was shown to be critical for muscle pathogenesis. These results indicate that *f-ATXN1^146Q/2Q^,* along with *f-ATXN1^146Q/2Q^*;*ACTA1-Cre*, provides an excellent experimental platform for elucidating the molecular aspects of ATXN1[146Q]-induced muscle pathogenesis.

In conclusion, we demonstrate the utility of the *f-ATXN1^146Q/2Q^* conditional mouse model in linking pathogenesis in specific anatomical regions/cell populations with SCA1 phenotypes. The results reveal that neural and peripheral pathological effects of expanded ATXN1 contribute independently to disease presentation. This finding has important implications for design and administration of optimal SCA1 therapeutics, especially in juvenile cases or late in disease for adult-onset cases. Notably, we show that the rotarod motor performance deficit is more complex in that, while initially driven by pathology of cerebellar Purkinje cells, pathology in striatal MSNs might contribute to this phenotype at late stages of disease. Lastly, data presented provide strong evidence of muscle-specific pathology in *f-ATXN1^146Q/2Q^* mice. Thus, an ideal SCA1 therapeutic needs to subdue mutant ATXN1 toxicity in the CNS and peripheral muscle.

## Methods

### Sex as a biological variable

This study examined male and female mice, and similar results were obtained for both sexes.

### Mice

All mice were housed and managed by Research Animal Resources under specific pathogen–free conditions in an Association for Assessment and Accreditation of Laboratory Animal Care International approved facility. The mice had unrestricted access to food and water except during behavioral testing. In all experiments, equal numbers of male and female mice were used. All mice were age matched within experiments, and littermate controls were used when possible. All mice were maintained on a C57BL/6N genetic background. *WT-Atxn1^2Q/2Q^* (C57BL/6J) mice, *ACTA1-Cre/HSACre79* (B6.Cg-Tg[ACTA1-Cre]79Jme/J, RRID:IMSR_JAX:006149) mice, and *Nestin-Cre* (B6.Cg-Tg[Nes-Cre]1Kln/J, RRID:IMSR_JAX:003771), *tdTomato* (B6N.129S6-Gt[ROSA]26Sor^tm1[CAG–tdTomato*,–EGFP*]Ees^/J, RRID:IMSR_JAX:023537) mice were obtained from The Jackson Laboratory. The *Rgs9-Cre* mice were a gift from X. William Yang (UCLA, Los Angeles, California, USA).

### Generation of f-ATXN1146Q/2Q mice

#### Embryonic stem cell culture.

C57BL/6N-PRX-B6N mouse embryonic stem (mES) cells were purchased from The Jackson Laboratory (012448, donating investigator Robin Wesselschmidt, Primogenix Inc.). Cells were maintained at 37°C at 7.5% CO_2_ in IMDM (Gibco, 12440-053, Iscoves DMEM) containing 20% ESC qualified FBS (Cytiva, SH30071.03), 1× NEAA (Gibco, 11140-050), 2 mM L-glutamine (Gibco, A2916801), 1× penicillin/streptomycin (Gibco, 15140-122), 0.2 mM β-mercaptoethanol (MilliporeSigma, M3148-100ML), 1,000 U/mL ESGRO-mouse LIF (MilliporeSigma, ESG1107). Cells were grown on gelatin-coated dishes containing irradiated mouse embryonic fibroblasts (iMEF; Thermo Fisher Scientific, A34180 and A34963). 

#### mES cell transfections, selection, analysis and expansion.

Genomic recombination was enhanced by Cas9-CRISPR cleavage at a site 5′ of the mouse *Atxn1* exon 7 (5′-TAAGCGGCTGTCTTGACCAC-3′) and a site 3′ of the *Atxn1* polyA site (5′-TAAGCTGTGGTTGCTTGAGC-3′) 19.6 kb from the first cleavage site. These sgRNA targets were cloned into pSpCas9(BB)-2A-Puro (AddGene plasmid ID 48139) as described previously (32). mES cells were trypsinized with 0.05% trypsin, plated onto gelatin-coated (no iMEF) 6-well dishes from a 90% ESC confluent p60 dish at a 1:6 dilution, and transfected with the pair of sgRNA/Cas9 plasmids and a repair template that inserted between the 2 cleavage sites a puromycin cassette flanked by FRT sites and an adjacent, promoterless neomycin gene that ended in a Lox site (Figure 1B). The next day, the ESC were transferred to 10 cm, gelatin-coated iMEF-puroR plates. Cells were allowed to recover for 24 hours. Puromycin (1.4 μg/mL) was added on day 2 and 3, with no puromycin on day 4. Puromycin (1.5 μg/mL) was added again on days 5–7. On day 8, selected clones were picked onto 96-well gelatin iMEF plates. When cells were 80%–90%confluent, ES cells were frozen in duplicate 96-well format (80% compete media, 10% additional ES FBS, 10% DMSO) and a third onto 48-well gelatin-only plate, in which cells were allowed to expand and were harvested for DNA analysis of each clone by PCR with the following primers: i6Atxn1 Junction (F1: 5′-ACACGTGGCTGCAATTTGTC-3′; R1: 5′-GTAACGCGCTTGCTGCTTG-3′) and 3′ of Atxn1 Junction (F1: 5′-AGCGTATCCACATAGCGT-3′; R1: 5′-CTTGCCCATTGCATACCAGG-3′). The puromycin cassette in clone 40 (C40) from this first transfection was replaced by human genomic DNA syntenic to the deleted mouse genomic sequences by flippase (Flp) recombinase using the approach as described (33). This 31 kb of human sequence (BAC RP11-413J6) extends from sequences 5′ of human ATXN1 exon 8 (5′-TAATGTTACACCAGGCTAAA-3′) to a sequence 3′ of the human ATXN1 polyA site (5′-AGGTGGAATCCCCTGCACCC-3′) and is flanked by FRT sites, a Lox sequence just 3′ of the 5′ FRT site; contains a 146Q expanded CAG repeated in exon 8; and, at the 3′ end, has a promoter-FRT cassette that drives expression of the neomycin resistance gene upon recombination into the modified mES cell. After transfection, mES cells were transferred to 10c m, gelatin-coated iMEF-Neo plates. Cells were allowed to recover for 48 hours and were then selected with 125 μg/mL of G418 (50 mg/mL, Thermo Fisher Scientific) for 7 days. Selected clones (13 total) were picked and expanded. The clones were frozen with 80% complete media, 10% additional ES-FBS, and 10% DMSO. PCR analyses were performed with the following primers: i6Atxn1 junction (F1: 5′-ACACGTGGCTGCAATTTGTC-3′; R2: 5′-GGATGGCTCTGATTTTAGTCTG-3′) and NeoR junction (F1: 5′-ACGAGCCTTCATAGCATCCG-3′; R1: 5′-GGATGGCTCTGATTTTAGTCTG-3′). C2 and C10 were correct for all assays and were sequence verified and further expanded. Chimeric mice were generated by injection of C2 and C10 ESC into blastocysts and backcrossed with C57BL/6J.

### Genotyping

PCR was performed with the following primers (Integrated DNA Technologies) to determine which animals have an expanded *f-ATXN1^146Q/2Q^* allele: ATXN1-146Q repeat forward (5′-CAACATGGGCAGTCTGAG-3′) and ATXN1-146Q repeat reverse (5′-GTGTGTGGGATCATCGTCTG-3′) To assess recombination by Cre the following primer set was used: recombination forward (5′-GGGAATGGTACCAACCTT TCTG-3′) and recombination reverse (5′-GTAGAACCCCAGACCCTCGT-3′). Cre F oIMR 1084 (5′-GCGGTCTGGCAGTAAAAACTATC-3′) and Cre R oIMR 1085 (5′-GTGAAACAGCATTGCTGTCACTT-3′) were used to genotype all Cre lines.

### Analyses of CAG instability

Genomic DNA was extracted from dissected striatum, medulla, cortex, hippocampus, and cerebellum using the Qiagen DNeasy Blood & Tissue kit according to the manufacturer’s protocol. Tail DNA was prepared using Wizard SV Genomice DNA kit (Promega, A2360). The *ATXN1* CAG repeat was PCR amplified using 1 μM each forward (6-FAM 5′-CAGAGTGGAATAGGCCTCCA-3′) and reverse (5′-TGGACGTACTGGTTCTGCTG-3′) human *ATXN1*-specific primers, 10 μL 2× Promega GoTaq Colorless Master Mix and 4 μL betaine in a total reaction volume of 20 μL. Cycling conditions were 95°C for 3 minutes, 32 cycles of 95°C for 1 minute, 58°C for 1 minute, and 72°C for 1 minute, followed by 72°C for 5 minutes. PCR products were run on the Applied Biosystems 3730xl DNA Analyzer using GS500LIZ internal size standard and analyzed using GeneMapper v5. Repeat size of the modal allele was inferred based on the size of the PCR product in base pairs. Expansion indices were calculated from GeneMapper peak height data as described previously (34) using a 5% relative peak height threshold. These were calculated relative to the modal allele of the more stable striatal peak, which was typically the lowest modal allele length among all tissues at 35 weeks and assumed to be the closest approximation to the inherited repeat length.

### Western blot

Cerebellum, brain stem, and cerebral cortex were collected from *WT-Atxn1^2Q/2Q^*, *f-ATXN1^146Q/2Q^*, and *Atxn1^175Q/2Q^* mice at 2 weeks of age. Samples were homogenized using a tissue grinder in 500 μL of Tris Triton lysis buffer (50 mM Tris [pH 7.5], 100 mM NaCl, 2.5 mM MgCl_2_, 0.5% Triton X-100) that included MilliporeSigma protease inhibitors II and III and a Roche Complete Mini Protease inhibitor tablet. Homogenized samples were shaken at 1500 rpm at 4°C for 1 hour, frozen, and thawed in liquid nitrogen and 37°C water bath 3 times; it was then centrifuged at 21,000*g* for 10 minutes at 4°C. Samples containing 30 μg total protein were boiled in Laemmli loading buffer and run on a 4%–20% Bio-Rad precast gel. Protein was transferred to a nitrocellulose membrane using the Bio-Rad Trans-Blot Turbo system. Blots were cut at approximately 75 kDa and blocked overnight at 4°C in 5% milk phosphate-buffered saline with 0.1% Tween 20 (PBST). Blots were probed overnight at 4°C 1:2,500 with the ATXN1 antibody 11750 (35) or 1:10,000 with α-tubulin antibody (MilliporeSigma, T5168) diluted in 5% milk PBST. Blots were washed 3 times with PBST. ATXN1 blots were then placed in 5% milk PBST plus 1:2,500 rabbit specific HRP antibodies (GE Healthcare, NA934V), while α-tubulin blots were placed in 5% milk PBST plus 1:10,000 mouse specific HRP antibodies (GE Healthcare, NXA931V) at room temperature for 4 hours. Blots were washed 3 times with PBST, and then ATXN1 blots were washed with Super Signal West Dura (Thermo Fisher Scientific) while α-tubulin blots were washed with Super Signal West Pico (Thermo Fisher Scientific) detection reagents. Blots were imaged on an ImageQuant LAS 4000. For the DARPP-32 Western blotting, striatal tissue was homogenized in sample buffer as above, blotted, and probed with rat monoclonal anti–DARPP-32 (R&D Systems, MAB4230) at 1:1,000 and anti–rat IgG HRP secondary at 1:2,500 (GE Healthcare, NA935).

### qPCR

One half of each brain region or tissue from each mouse was homogenized in 500 mL TRIzol Reagent (Thermo Fisher Scientific, 15596026). RNA isolation was done per the manufacturer’s instructions. cDNA was synthesized in duplicate using 500 ng RNA in 10 μL iScript Advanced cDNA Synthesis Kit (Bio-Rad, 172-5038). Reactions were diluted 1:5 in water. qPCR was done using 2 μL diluted cDNA in 10 μL Roche Probes Master (04707494001) reactions on a Roche 480 Lightcycler. Target gene and reference gene reactions were amplified in separate wells under cycling conditions of 95°C for 10 seconds and 60°C for 10 seconds for 35 cycles. Primers used include ATXN1 forward (5′-AGAGATAAGCAACGACCTGAAGA-3′) and ATXN1 reverse (5′-CCAAAACTTCAACGCTGACC-3′) with Roche probe 67 (04688660001). NMJ QPCR was done using 2 μL diluted cDNA in 10 μL Roche Sybr Green (04707516001) reactions under cycling conditions of 95°C for 10 seconds, 50°C for 10 seconds, and 72°C for 10 seconds for 45 cycles. Primers used include Chrna1 forward (5′-CATCGAGGGCGTGAAGTACA-3′), Chrna1 reverse (5′-ATTCCTCAGCGGCGTTATTG-3′), Musk forward (5′-TGAGAACTGCCCCTTGGAACT-3′), and Musk reverse (5′-GGGTCTATCAGCAGGCAGCTT-3′). Reference genes from Roche (Proprietary sequence) include mGapdh (5046211001) and mActb (05046190001). PrimeTime Hprt reference gene from IDT was Mm.PT.39a.22214828. Relative expression was determined by simultaneous comparison to 3 reference genes — Gapdh, Actb, Hprt — using RefFinder 2020.10.20-2024.02.07 (36, 37). Quantitation cycle (Cq) values were determined using the Roche second derivative maximum calculation. Relative quantification was done using standard 2^ΔΔCq^.

### Total mouse/human qPCR assay

Total *ATXN1* RNA levels were measured using primers and probes that measure both human and mouse RNA levels in the same reaction. The assay was designed with a forward primer that binds to Exon 6 of the mouse sequence, which is common for all genotypes (mAtxn1 forward, 5′-AAGAAAGACACCACCAGAACC-3′). The reverse primer was designed to bind to an identical sequence shared by mouse exon 7 and human exon 8 (m+hATXN1 reverse, 5′-GATTTCTGTAGGGGATCCAGGC-3′). This will generate 2 highly similar but unique amplicons using a single set of primers. Quantitation of the 2 amplicons will be achieved by utilizing 2 probes. Probes were designed to bind to unique sequences within the amplicons conferring specificity for human or mouse sequence: mAtxn1 Ex6-Ex7 (56-FAM/CCACTGCCA/ZEN/GCCTAAAGAACCCA/3IABkFQ) or hAtxn1 Ex8 (5HEX/CCAGAGCTG/ZEN/C TGTTGGCGGATTGTA/3IABkFQ). qPCR was optimized for primer concentration and probe concentration and checked for reaction efficiency. qPCR efficiency of amplification for mouse reaction was 1.926, and for human reaction, it was 1.925.

#### CT scan.

Mice were anesthetized with 2.5% isoflurane, and CT scans were performed using a Sophie G8 μPET/μCT imaging system (PerkinElmer). The x-ray source for the CT scans were set to 59 kVp and 100 μA, and the resulting voxel size was 200 μm isotropic. Three-dimensional CT images were analyzed using Imaris 9.8 (Oxford Instruments). KI was determined based on where the distance was calculated from a horizontal line drawn from the center of the C7 vertebrae to the center of the pelvis, and a vertical line was drawn from the apex spine curvature to the intersection of the horizontal line (21). Measurement points were manually added in 3-dimensional spaces using the Imaris spots module.

### Behavior methods

A behavioral battery was used to assess neuromotor and neurocognitive function in individual animals over time. Testing was repeated at 6, 12, 18, 26, 30, and/or 30 weeks of age.

#### Rotarod.

A rotarod apparatus (Ugo Basile) was used to assess motor coordination and balance. Testing occurred over 4 consecutive days, and each test day consisted of 4 individual trials (~15-minute intertrial interval) where mice were placed on a rotating rod that accelerated from 5 to 50 rpm in 1-rpm steps over the course of a 5-minute interval (~7-second ramp). The trial ended when mice fell from the rod, when they failed to continuously walk on the rod (held on to the rod instead of walking for 2 full 360° rotations), or after the 5-minute maximum trial time was reached. The apparatus was cleaned with 70% ethanol between animals and between each trial. The latency to fall (seconds) was averaged across the 4 trials in individual animals for analysis.

#### Barnes maze.

A Barnes maze apparatus (San Diego Instruments) was used to assess spatial learning and memory (38). The apparatus consisted of a white circular ABS plastic arena (36-inch diameter, 36 inches high) with twenty 2-inch holes evenly spaced along the perimeter. One of the holes contained a recessed target box that allowed the mice an escape from the open space of the arena. Contextual markers were located on the walls around the maze to provide spatial cues, and the apparatus was brightly illuminated (~250 lux) during testing. Training consisted of 4 trials a day (~15-minute intertrial interval) for 4 days, and a trial began by placing the mouse in the center of the arena and ended when the mouse entered the goal box or after the 3-minute maximum test time had elapsed. The location of the goal box remained the same across training trials. A probe test was conducted the following day in which the target hole was blocked off and mice were allowed to explore the arena during a single 90-second trial. The apparatus was cleaned with 70% ethanol between animals and between each trial. The latency (seconds) to enter the escape hole was averaged across the 4 trials in individual animals each day during training, and the time spent (seconds) in the goal versus the other 3 quadrants of the maze (+1, opposite, –1) was assessed for the probe test.

#### Open field.

An open-field arena was used to assess exploratory locomotor activity and consisted of a white rectangular box (20-inch width × 20-inch length × 10-inch height) illuminated by overhead LED lights (~150 lux). Mice were allowed to explore the open field arena for 30 minutes, and ANY-maze video tracking software was used to measure the total distance traveled (m) and average speed (m/sec) in individual animals. The apparatus was cleaned with 70% ethanol between animals.

#### Grip strength.

Grip strength testing was conducted using a digital meter (Bioseb). Mice were gently lifted by the tail, and the front paws were lowered onto a wire grid so that the front paws grabbed on; then, the mice were gently pulled back horizontally from the grip bar at a constant speed until the grip released. This was repeated for 4 trials, and grip strength (g) was averaged across the trials. Animal weights were used to calculate strength/weight ratios.

### Muscle strength assays

#### In vivo torque.

Mice were anesthetized with isoflurane, and maximal isometric torque of the anterior crural muscles was measured. Sterilized platinum needle electrodes were placed through the skin near the left common peroneal nerve and were connected to a stimulator (Models S48, Grass Technologies) and stimulus isolation unit (SIU5, Grass Technologies). The contractile function of the anterior crural muscles was assessed by measuring isometric torque (variable voltage [3–10 V], 150-ms train, and 0.1-ms pulses) every minute until peak torque was achieved.

#### Ex vivo muscle preparation.

EDL force production was assessed according to methods described previously (39).Briefly, mice were anesthetized with sodium pentobarbital (75–100 mg/kg body mass), and EDL muscles were excised and mounted in a 1.2 mL bath assembly with oxygenated (95%:5% O_2_/CO_2_) Krebs Ringer bicarbonate (Krebs) buffer maintained at 25°C. Muscles were adjusted to their anatomical optimal length (Lo) based on resting tension, with length being measured from the distal myotendinous junction to the proximal myotendinous junction. Muscles were equilibrated in the bath for 10 minutes before performing isometric contractions using maximal voltage (150 V) for 200 ms at 175 Hz every 2 minutes until peak isometric force was achieved.

### Immunofluorescence

TA muscles from each mouse line were prepared in melting isopentane for 30 seconds, and 10 μm transverse cryosections were obtained (Leica CM3050 S). Sections were fixed in acetone at –20°C for 15 minutes and subsequently washed 3× in PBS before being blocked in 5% goat serum for 30 minutes at room temperature. Sections were then incubated for > 1 hour in primary antibody (rat monoclonal anti–Laminin, 1:500, MilliporeSigma, L06631) at room temperature, washed 3× with PBS, and incubated with Alexa Fluor 488 goat anti–rat IgG (Thermo Fisher Scientific, A-11006) secondary (1:1,000) for 30 minutes at room temperature. Finally, sections were washed 3× in PBS and mounted in ProLong Gold Antifade with DAPI to visualize nuclei (Thermo Fisher Scientific). Images were acquired on a Leica DM5500 B microscope equipped with a Leica HC PLAN APO 10× objective and stitched together with LASX software (Leica) to allow visualization of the entire TA. SMASH – semi- automatic muscle analysis using segmentation of histology software was used to analyze and quantify centrally located nuclei, fiber number, and fiber size (40).

### Statistics

All data were analyzed using GraphPad Prism software (v10.0). All data were checked for normality and met assumptions for using parametric statistical tests. For multigroup comparisons, 1-way or 2-way ANOVA were performed. When working with repeated-measures data, sphericity was not assumed; thus, a Geisser-Greenhouse correction was used. When ANOVA findings were significant (*P* < 0.05), the analysis was followed by multiple comparisons testing using Tukey’s (all pairwise comparisons, > 2 groups), Šídák’s (all pairwise comparisons, 2 groups), or Dunnett’s (pairwise comparisons back to 1 control group, > 2 groups) correction for multiple comparisons. Survival analysis, plotted as Kaplan-Meyer curves, was assessed using log-rank Mantel-Cox and Gehan-Breslow-Wilcoxon tests. Western blots were quantified using ImageQuant TL software (Cytiva). All data presented are shown as mean ± SEM. *P* < 0.05 was considered significant.

### Study approval

The University of Minnesota IACUC approved all animal use protocols. All mice were housed and managed by Research Animal Resources under specific pathogen–free conditions in an Association for Assessment and Accreditation of Laboratory Animal Care International approved facility.

### Data availability

Values for all data points in graphs are reported in the Supporting Data Values file.

## Author contributions

LD and HTO conceived the study and wrote the paper. LD, WMS, JME, MC, and HTO designed experiments and interpreted the data. KAB and MDK designed the strategy and generated the *f-ATXN1^146Q/2Q^* mouse model. YY performed embryo injections and implantations. OR and SS bred mice and managed the colony. LD, OR, and SS conducted the survival study and animal weight measurements. OR, EBL, and TNM performed behavioral assays. HK performed qPCR, Western blot analyses, and genotyping. PY, AS, and CAS performed dissection and recombination assay. UG and BO assessed the degree of Cre-mediated recombination. BO performed immunofluorescence and imaging. JSM developed and implemented kyphosis image analyses. WMS performed muscle histological and functional analyses. HPH generated the *Atxn1^175QK772T/2Q^* mouse model and performed statistical analyses. HYZ designed experiments and edited the manuscript. VCW, HTO, and LD designed the repeat instability experiment. ZNB performed the repeat instability experiment and analyzed the data. VCW and ZNB interpreted the data, and VCW contributed to the writing of the manuscript. All authors reviewed the manuscript and provided input. LD is listed as first author since she organized the data for the manuscript as well as led planning and performance of the experiments. LD, WMS, and KAB are listed as co–first authors; order of authorship was decided based on type of contribution. LD designed and oversaw the entire project, WMS designed and performed muscle pathology experiments, and KAB generated the *f-ATXN1^146Q/2Q^* mice. MDK and HTO jointly supervised this work.

## Supplementary Material

Supplemental data

Supporting data values

## Version 1 03/21/2024

In-Press Preview

## Version 2 05/08/2024

Electronic publication
