# Supplementary material for: Mapping SCA1 regional vulnerabilities reveals neural and skeletal muscle contributions to disease
Source: JCI Insight. 2024 Mar 21;9(9):e176057. doi: 10.1172/jci.insight.176057 (PMC11141930; doi:10.1172/jci.insight.176057)
Supplement: Supplemental data [file jciinsight-9-176057-s238.pdf]

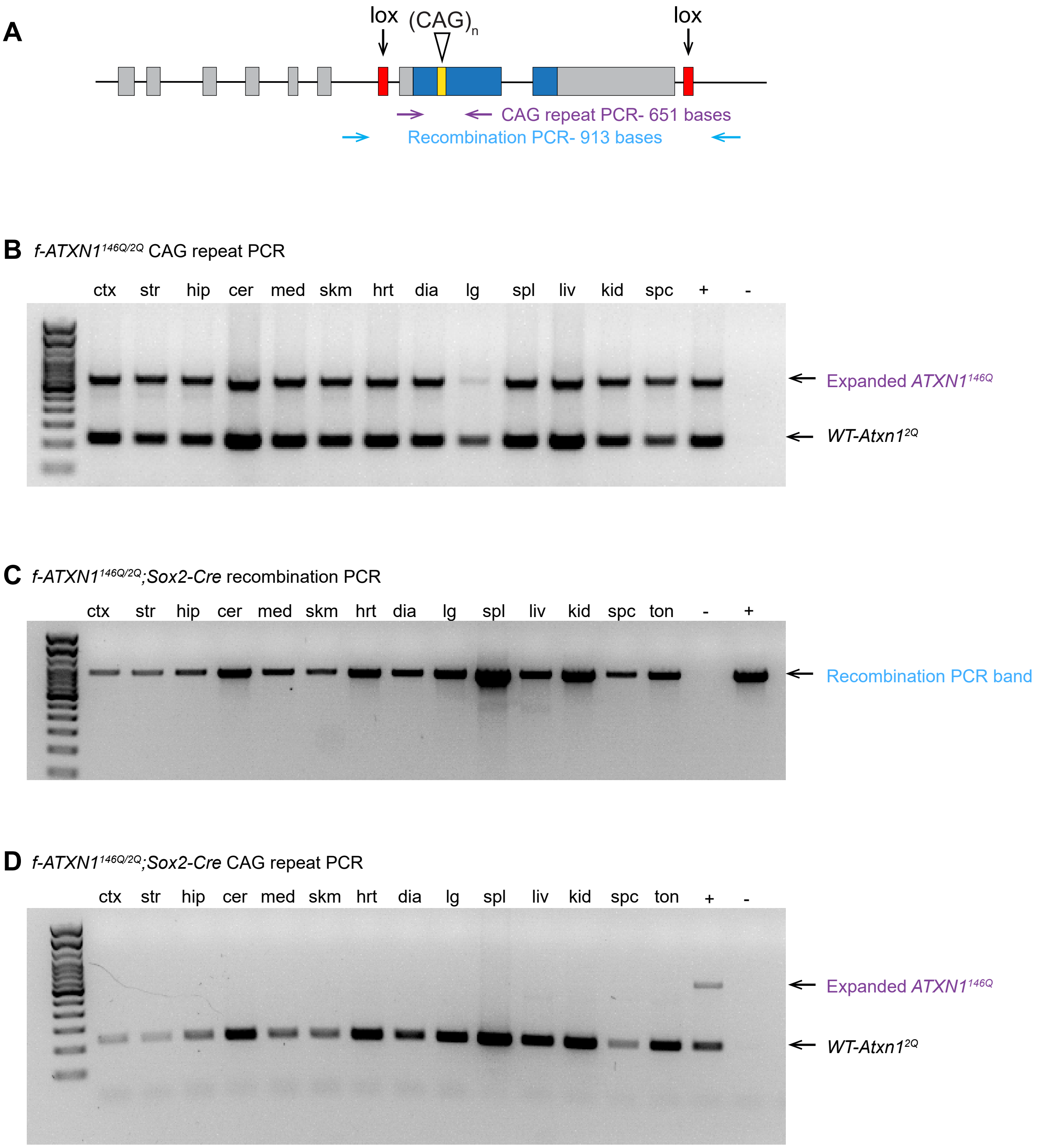

Figure S1. *f-ATXN1*$^{146Q/2Q}$ diagram and PCR.
(A) Schematic of *f-ATXN1*^{146Q} allele into the mouse endogenous locus. **(B)** Repeat PCR in tissue DNA from the *f-ATXN1*$^{146Q/2Q}$. Lanes: cortex (ctx), striatum (str), hippocampus (hip), cerebellum (cer), medulla (med), skeletal muscle (skm),heart (hrt), diaphragm (dia), lung (lg), spleen(spl), liver (liv), kidney (kid), spinal cord (spc), repeat positive control (+), recombination control (-). **(C)** Recombination PCR and **(D)** repeat PCR in tissue DNA from *f-ATXN1*$^{146Q/2Q}$*;Sox2-Cre* mouse. Lanes: cortex (ctx), striatum (str), hippocampus (hip), cerebellum (cer), medulla (med), skeletal muscle (skm),heart (hrt), diaphragm (dia), lung (lg), spleen(spl), liver (liv), kidney (kid), spinal cord (spc), tongue (ton) repeat positive control , recombination control .

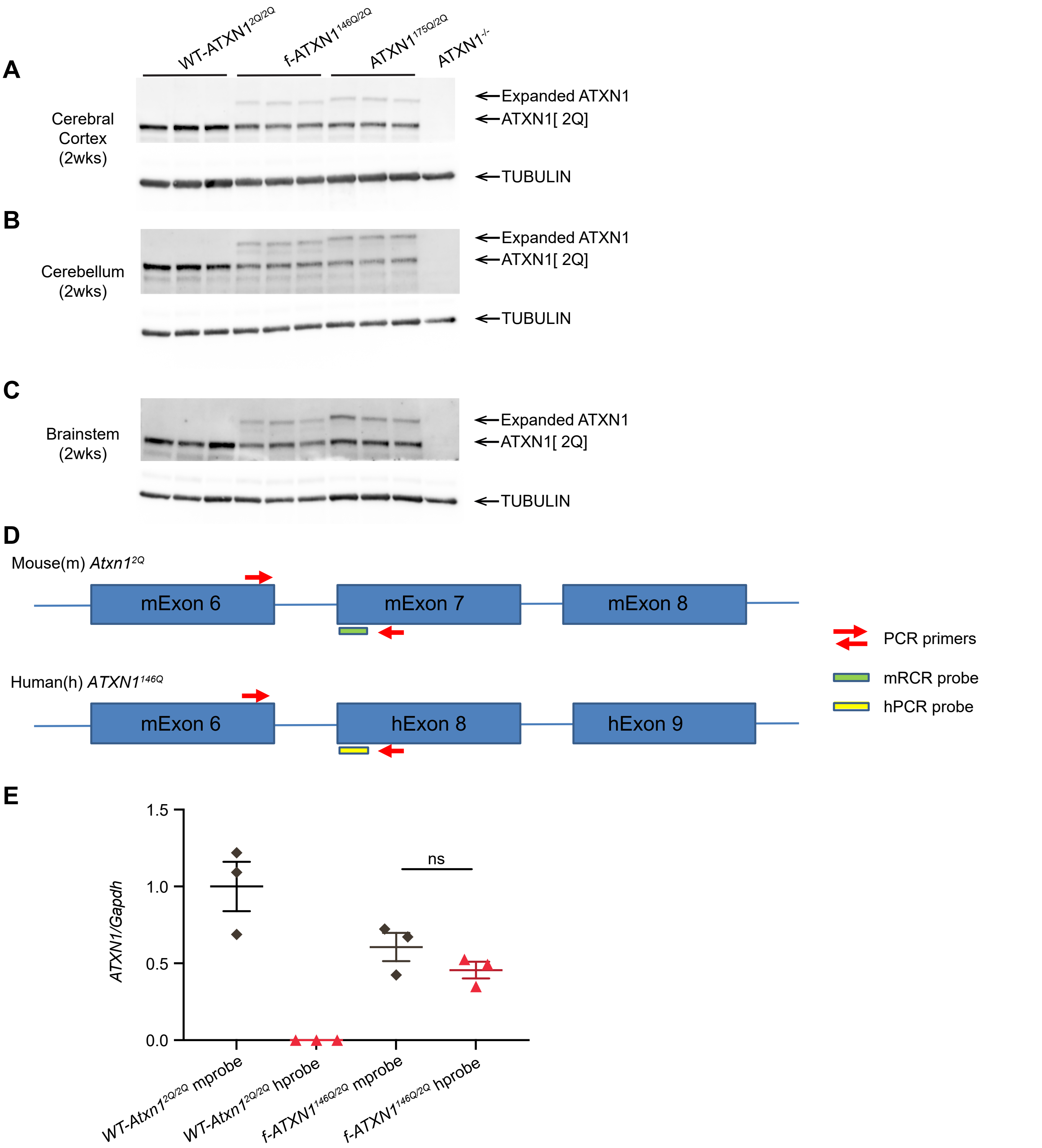

Figure S2. *f-ATXN1$^{146Q/2Q}$* mice express equal protein and RNA as endogenous gene.
(A-C) Western blot of expanded ATXN1 protein in *f-ATXN1$^{146Q/2Q}$* and *Atxn1$^{175Q/2Q}$* in 2-week-old mice from cortex, cerebellum, and brainstem tissue. **(D)** Schematic of the RT-QPCR reaction designed to quantify either *ATXN1^{146Q}* (human specific) or *WT-Atxn1^{2Q}* (mouse specific) RNA expression using identical primer sequences and unique probe sequences. **(E)** Relative expression of *ATXN1* in *f-ATXN1$^{146Q/2Q}$* mice and *Atxn1* in *WT-Atxn1$^{2Q/2Q}$* mice. Unpaired t test shows no signifigance between mouse and human transcript ($p>0.05$).

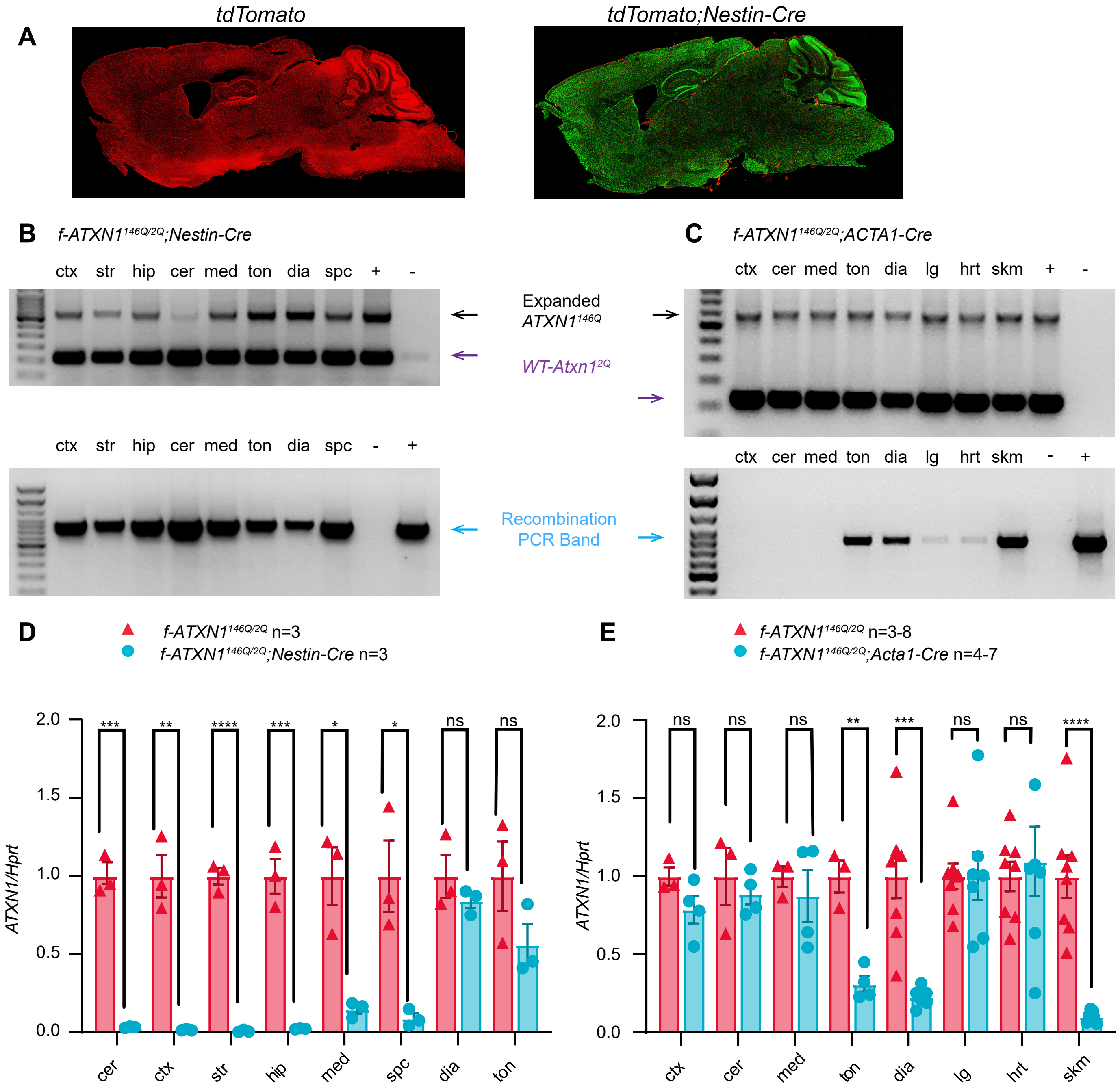

Figure S3. *ATXN1* expression in *f-ATXN1$^{146Q/2Q}$;Nestin-Cre* and *f-ATXN1$^{146Q/2Q}$;ACTA1-Cre* mice.
(A) Representative images showing Cre-recombination from a *tdTomato;Nestin-Cre* reporter mouse (green). **(B)** Repeat and recombination PCR in tissue DNA from the *f-ATXN1$^{146Q/2Q}$;Nestin-Cre*. Lanes: cortex (ctx), striatum (str), hippocampus (hip), cerebellum (cer), medulla (med), tongue (ton), diaphragm (dia), spinal cord (spc) and controls. **(C)** *f-ATXN1$^{146Q/2Q}$;ACTA1-Cre* mouse. Lanes: cortex (ctx), cerebellum (cer), medulla (med), tongue (ton), diaphragm (dia), lung (lg), heart (hrt), skeletal muscle (skm) and controls. **(D)** RT-QPCR of *ATXN1-146Q RNA* knockdown in tissue from *f-ATXN1$^{146Q/2Q}$;Nestin-Cre* and **(E)** *f-ATXN1$^{146Q/2Q}$;ACTA1-Cre* mice. Unpaired t test for each tissue pair. Significance of results is denoted as * ($p<0.05$), ** ($p<0.01$) and *** ($p<0.001$).

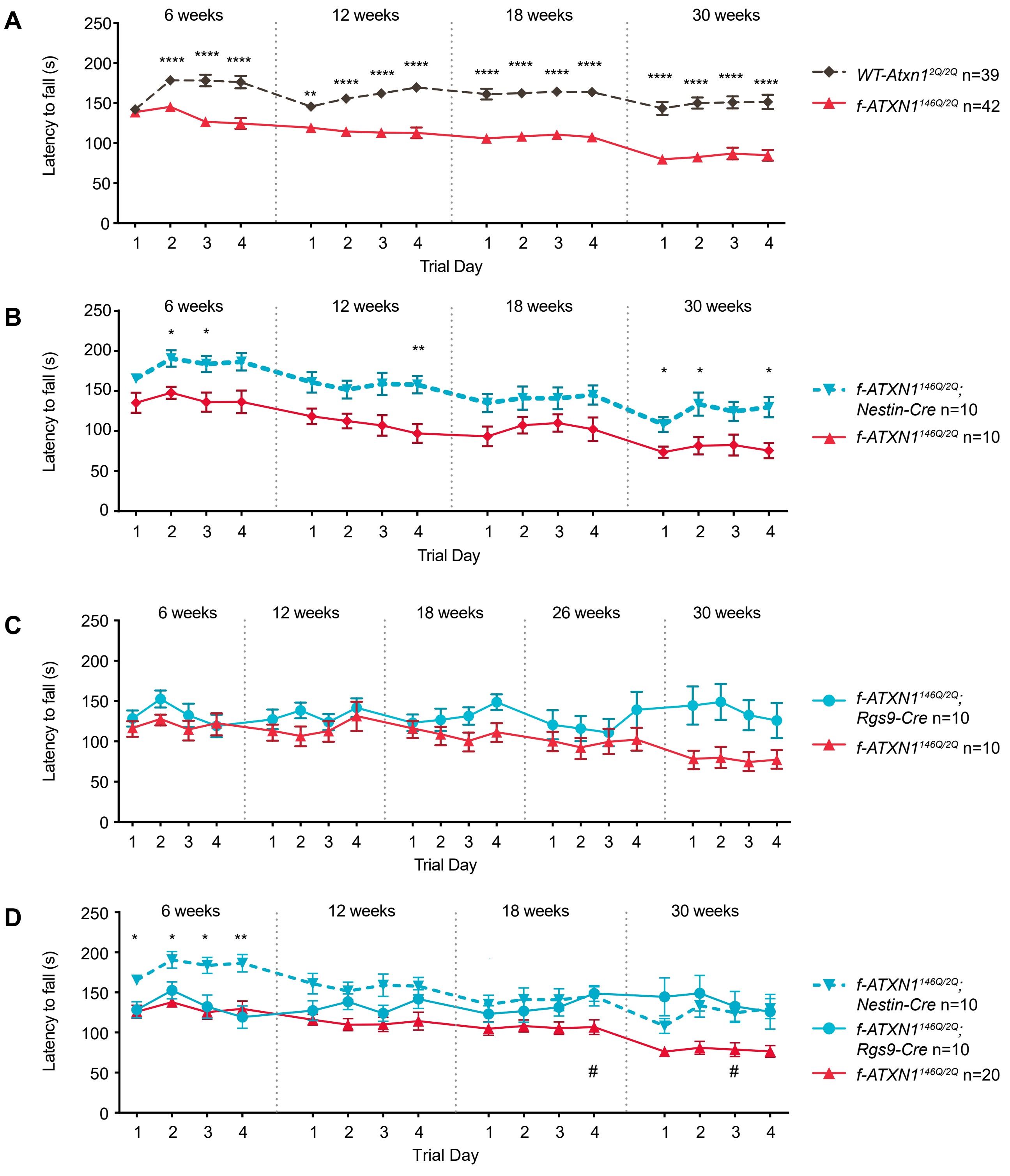

Figure S4. CNS regional contribution to progressive motor performance deficit in *f-ATXN1$^{146Q/2Q}$* mice.
(A) Rotarod assessment of combined data for *WT-Atxn1$^{2Q/2Q}$* and *f-ATXN1$^{146Q/2Q}$* mice across 4 trial days at 6,12,18, and 30 weeks-of-age. **(B)** Rotarod assessment of *f-ATXN1$^{146Q/2Q}$* and *f-ATXN1$^{146Q/2Q}$;Nestin-Cre*. **(C)** Rotarod assessment of *f-ATXN1$^{146Q/2Q}$* and *f-ATXN1$^{146Q/2Q}$;Rgs9-Cre*. **(D)** Rotaroad comparison of *f-ATXN1$^{146Q/2Q}$;Nestin-Cre* and *f-ATXN1$^{146Q/2Q}$;Rgs9-Cre* with combined *f-ATXN1$^{146Q/2Q}$*. RM two-way ANOVA with Geisser-Greenhouse correction and Tukey post hoc test. * denotes signifcance between *f-ATXN1$^{146Q/2Q}$;Nestin-Cre* and *f-ATXN1$^{146Q/2Q}$;Rgs9-Cre* and # denotes when both *f-ATXN1$^{146Q/2Q}$;Nestin-Cre* and *f-ATXN1$^{146Q/2Q}$;Rgs9-Cre* are signifcantly improved compared to *f-ATXN1$^{146Q/2Q}$*. **(A-C)** RM two-way ANOVA with Geisser-Greenhouse correction and Šídák post hoc test. No significant difference in **(C).** Significance of results is denoted as * (p<0.05), ** (p<0.01), *** (p<0.001), and **** (p<0.0001).

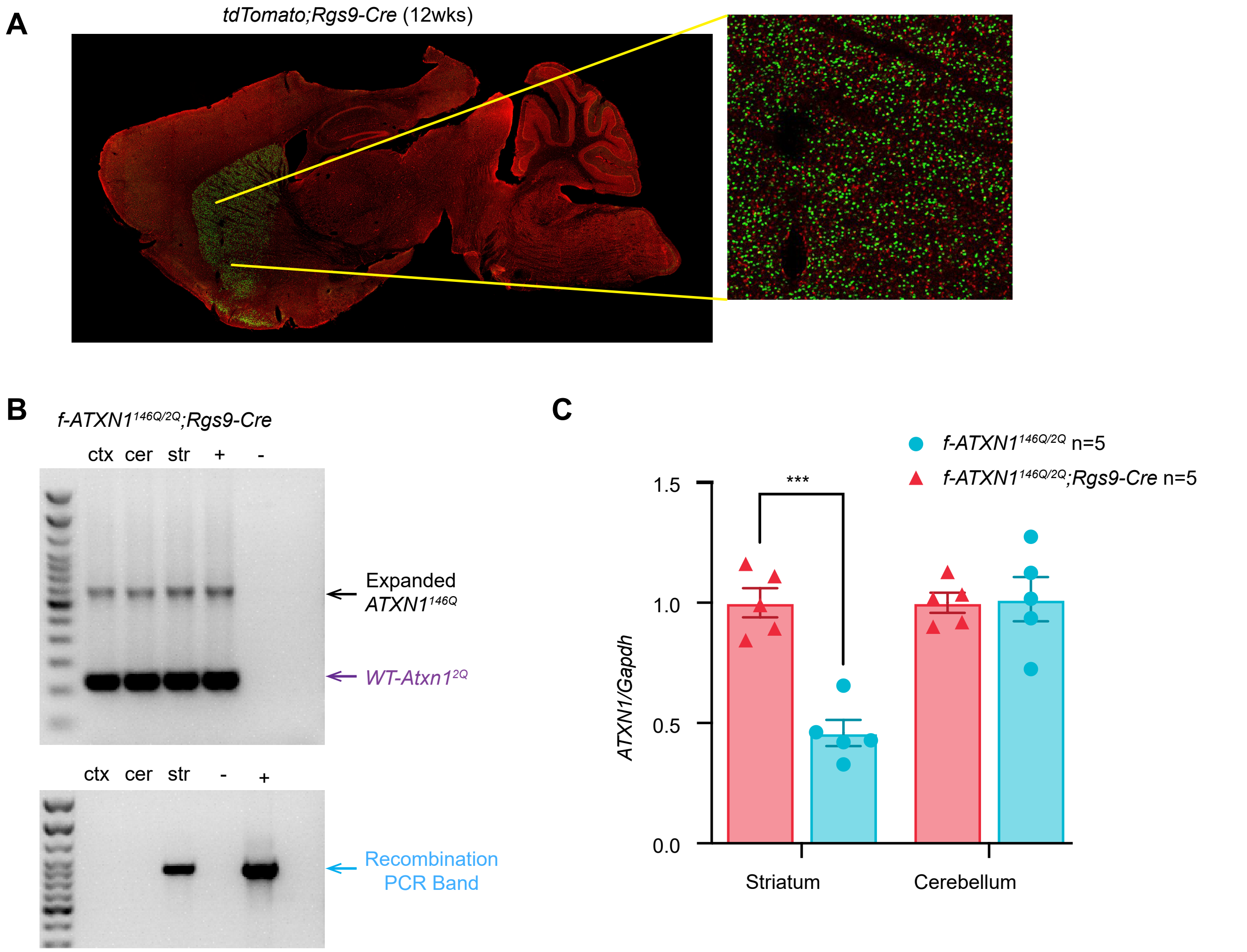

Figure S5. ATXN1 expression in *f-ATXN1$^{146Q/2Q}$;Rgs9-Cre* mice.
(A) Representative images showing Cre-recombination in the striatum (green) from a *tdTomato;Rgs9-Cre* reporter mouse. **(B)** Repeat and recombination PCR in tissue DNA from the *f-ATXN1$^{146Q/2Q}$;Rgs9-Cre*. Lanes: cortex (ctx), cerebellum (cer), striatum (str) and controls. **(C)** RT-QPCR of *ATXN1* knockdown in tissue from 35 wk *f-ATXN1$^{146Q/2Q}$;Rgs9-Cre*. Unpaired t test. Significance of results is denoted as * (p<0.05), ** (p<0.01), and *** (p<0.001).

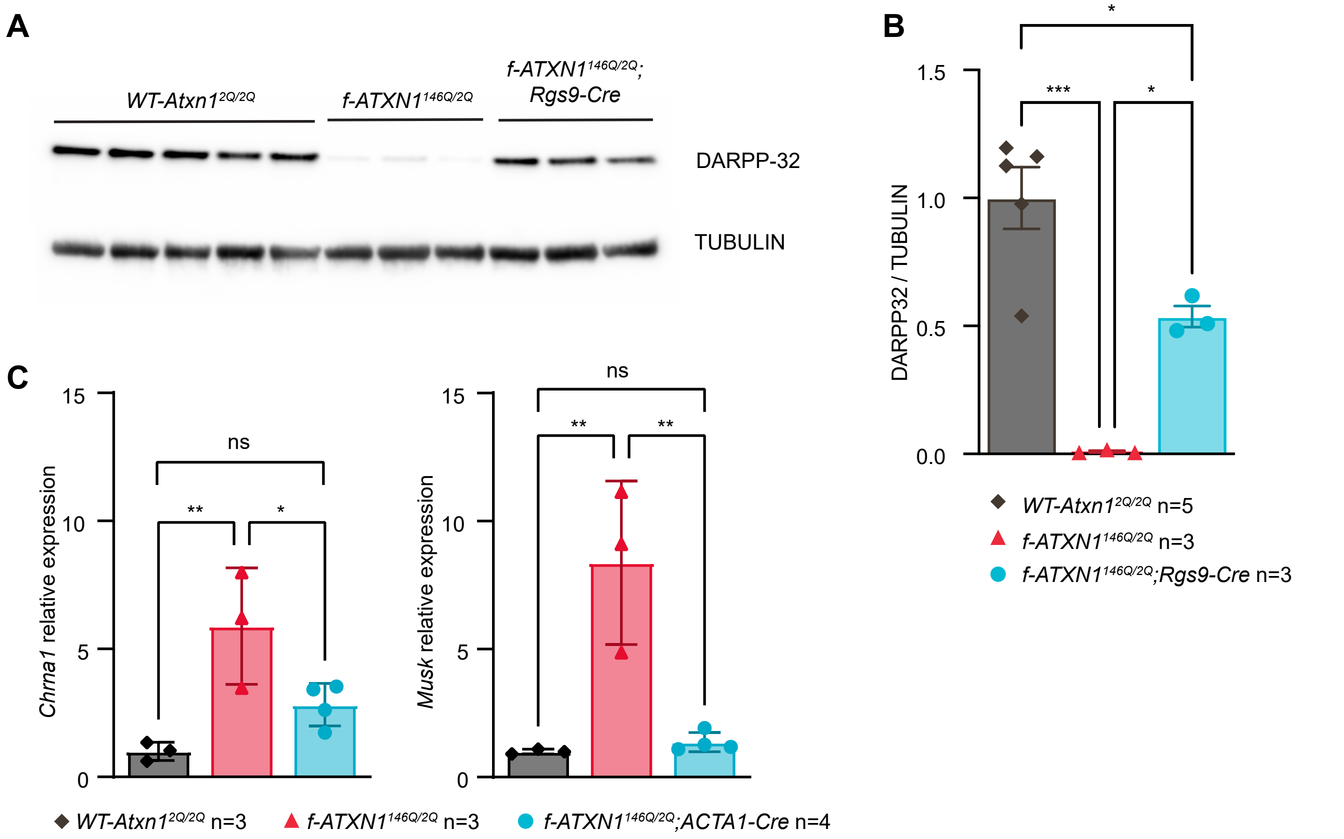

Figure S6. SCA1-like phenotypes corrected in *f-ATXN1*$^{146Q/2Q}$*;ACTA1-Cre* and *f-ATXN1*$^{146Q/2Q}$*;Rgs9-Cre*.
(A) Western blot quantification of DARPP-32 protein from striatum of *WT-Atxn1*$^{2Q/2Q}$, *f-ATXN1*$^{146Q/2Q}$ and *f-ATXN1*$^{146Q/2Q}$*;Rgs9-Cre* mice. **(B)** Quantification of DARPP32 protein relative to TUBULIN. One-way ANOVA with Tukey post hoc test. **(C)** Relative expression of *Chrna1* or *Musk* to the average of *Gapdh* and *Actb* in 8 wk quadricep RNA from *WT-Atxn1*$^{2Q/2Q}$ and *f-ATXN1*^{146Q2Q}*;ACTA1-Cre*. One-way ANOVA with Tukey post hoc test. Significance of results is denoted as * ($p<0.05$), ** ($p<0.01$), *** ($p<0.001$).

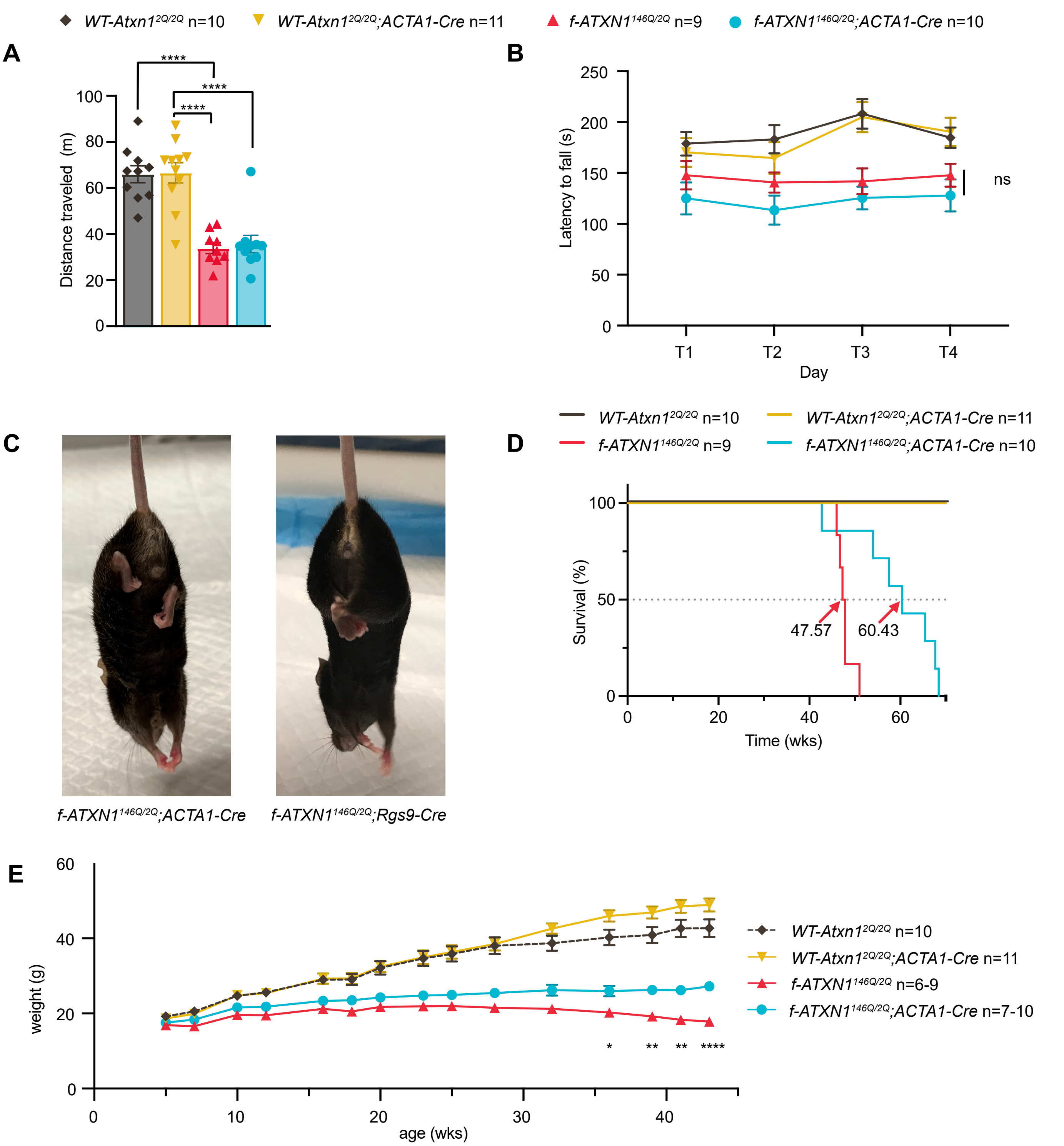

Figure S7. SCA1-like phenotypes in *f-ATXN1$^{146Q/2Q}$;ACTA1-Cre* mice.

(A) Distance traveled in open field at 12 weeks-of-age. One-way ANOVA with Tukey post hoc test. **(B)** Rotarod assessment of all 4 genotypes at 12 weeks. No significant difference between *f-ATXN1$^{146Q/2Q}$;ACTA1-Cre* and *f-ATXN1$^{146Q/2Q}$*. RM two-way ANOVA Geisser-Greenhouse correction with Tukey's Post hoc test. **(C)** Clasping phenotype in 35 wk *f-ATXN1$^{146Q/2Q}$;ACTA1-Cre*, and *f-ATXN1$^{146Q/2Q}$;Rgs9-Cre*. **(D)** Mouse survival plotted as Kaplan-Meyer curves with median lifespan labeled for each genotype. Log-rank (Mantel Cox) ****(p<0.0001) and Gehan-Breslow-Wilcoxon ****(p<0.0002). **(E)** Body weight measurements between 5 and 45 weeks of age. Significant difference between *f-ATXN1$^{146Q/2Q}$* and *f-ATXN1$^{146Q/2Q}$;ACTA1-Cre* starting at 36 wks. RM two-way ANOVA Geisser-Greenhouse correction with Tukeys Post hoc test. Significance of results is denoted as * (p<0.05), ** (p<0.01), *** (p<0.001) and ****(p<0.0001).
